# Autotaxin–lysolipid signaling suppresses a CCL11–eosinophil axis to promote pancreatic cancer progression

**DOI:** 10.1038/s43018-023-00703-y

**Published:** 2024-01-09

**Authors:** Sohinee Bhattacharyya, Chet Oon, Luis Diaz, Holly Sandborg, Erin S. Stempinski, Michelle Saoi, Terry K. Morgan, Claudia S. López, Justin R. Cross, Mara H. Sherman

**Affiliations:** 1 Department of Cell, Developmental & Cancer Biology, Oregon Health & Science University, Portland, OR USA; 2 Cancer Biology & Genetics Program, Memorial Sloan Kettering Cancer Center, New York, NY USA; 3 Multiscale Microscopy Core Facility, Oregon Health & Science University, Portland, OR USA; 4 Donald B. and Catherine C. Marron Cancer Metabolism Center, Memorial Sloan Kettering Cancer Center, New York, NY USA; 5 Department of Pathology, Oregon Health & Science University, Portland, OR USA; 6 Department of Biomedical Engineering, Oregon Health & Science University, Portland, OR USA

**Keywords:** Cancer microenvironment, Pancreatic cancer, Tumour immunology, Cancer

## Abstract

Lipids and their modifying enzymes regulate diverse features of the tumor microenvironment and cancer progression. The secreted enzyme autotaxin (ATX) hydrolyzes extracellular lysophosphatidylcholine to generate the multifunctional lipid mediator lysophosphatidic acid (LPA) and supports the growth of several tumor types, including pancreatic ductal adenocarcinoma (PDAC). Here we show that ATX suppresses the accumulation of eosinophils in the PDAC microenvironment. Genetic or pharmacologic ATX inhibition increased the number of intratumor eosinophils, which promote tumor cell apoptosis locally and suppress tumor progression. Mechanistically, ATX suppresses eosinophil accumulation via an autocrine feedback loop, wherein ATX–LPA signaling negatively regulates the activity of the AP-1 transcription factor c-Jun, in turn suppressing the expression of the potent eosinophil chemoattractant CCL11 (eotaxin-1). Eosinophils were identified in human PDAC specimens, and rare individuals with high intratumor eosinophil abundance had the longest overall survival. Together with recent findings, this study reveals the context-dependent, immune-modulatory potential of ATX–LPA signaling in cancer.

## Abstract (web summary)

Sherman and colleagues show that ATX suppresses the accumulation of eosinophils in the tumor microenvironment of pancreatic cancer via suppression of CCL11 expression and find that ATX inhibition increases eosinophil influx, promoting tumor cell death.

## Main

The complexity of the pancreatic ductal adenocarcinoma (PDAC) microenvironment has presented a challenge to efforts aimed at better understanding the diverse cell types and acellular features therein and how they impact tumor progression. Increasingly detailed analyses of PDAC immune contexture have revealed cellular and spatial features associated with immune suppression and poor prognosis. Exclusion of leukocyte populations (for example, T cells and dendritic cells) that are necessary for adaptive immune recognition of cancer is a common feature of PDAC^1–4^. By contrast, PDAC is known for its recruitment of myeloid populations that possess immune-suppressive and tumor-promoting features, which in part determine treatment outcomes^5^. Further, myeloid cell phenotype is pliable such that infiltrating myeloid cells may be engendered with either tumor-promoting or tumor-suppressive features. Myeloid cells may support tumor progression by enabling many of the hallmarks of cancer, including the functions of arginase-1-expressing macrophages^6^ and granulocytes such as neutrophils^7,8^. However, myeloid cells can also be endowed with antitumor properties, including the capacity to directly mediate tumor cell killing^9^, and support productive T cell immunity^3,4^. Although granulocytes within the myeloid compartment remain largely understudied in PDAC, emerging evidence of the antitumor potential of eosinophils in other solid tumors^10^ motivates the investigation of these cells in PDAC.

The immune evasiveness of PDAC is multifactorial and is influenced by cancer cell-intrinsic and -extrinsic mechanisms. Genetic mouse models suggest that pancreatic tumors may be composed of cancer cell clones with vastly distinct immunogenicity and responsiveness to immunotherapy^11^. In these studies, clonal cell lines generated from tumors arising spontaneously in pancreas-specific *Kras*-mutant, *Trp53*-mutant (KPC) mice were found to produce tumors with either a strong or weak T cell infiltrate when transplanted into syngeneic mice. In addition, these clones responded differentially to immunotherapy, with strong responses observed for T cell-enriched tumors. However, after mixing of the cancer cell clones, the phenotype of T cell-poor tumors was shown to dominate, underscoring the need to identify and target immune-modulatory mechanisms derived from the neoplastic compartment.

Lysophosphatidic acid (LPA) mediates tissue remodeling and tumor progression by functioning as an autocrine motility factor, promoting cell proliferation and migration^12^. Signaling through the G-protein-coupled LPA receptors expressed by normal and malignant cell types, including PDAC cells, LPA activates mitogenic pathways, including RAS–ERK and PI3K^13^. This signaling mechanism has been implicated in the progression of several human cancer types, including colorectal, prostate and ovarian cancer^14–16^. Autotaxin (ATX) is the key LPA-producing enzyme in tissues and plasma^13^, as it hydrolyzes lysophosphatidylcholine to generate LPA via lysophospholipase D activity^13,17^. Although essential during embryonic development^13^, ATX activity is dispensable for adult organismal health^13^, highlighting its potential as a therapeutic target in cancer. We previously showed that ATX–LPA signals through LPA receptors on PDAC cells to promote mitogenic signaling and tumor growth^13^. However, as LPA also regulates inflammation and leukocyte trafficking in diverse tissue contexts^18–20^, we questioned the impact of ATX–LPA signaling on the immune microenvironment of PDAC. An ATX inhibitor, IOA-289, has recently entered a clinical trial for individuals with PDAC (NCT05586516) based on promising preclinical results in gastrointestinal tumor models^21–23^, and a deeper understanding of ATX function in PDAC may inform translational efforts. Beyond implications for PDAC biology, we reasoned that understanding the broad regulation of PDAC cellular composition by ATX–LPA signaling may lead to the identification of rationally designed combination therapies in the future.

## Results

### ATX signaling promotes pancreatic tumor progression

**Fig. 1:**
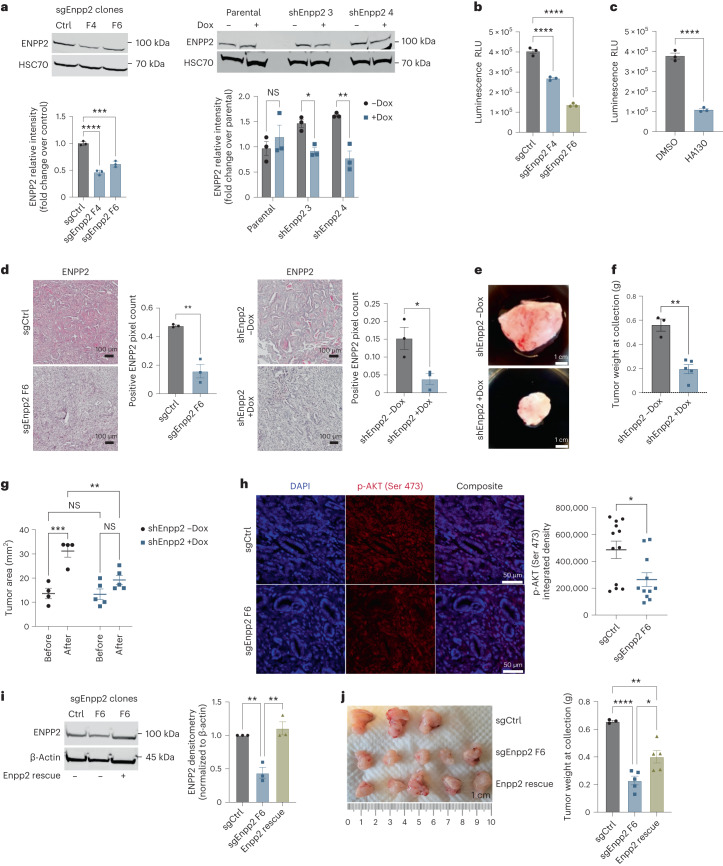
ATX signaling promotes pancreatic tumor progression. **a**, Western blots depicting ENPP2 (ATX) abundance in FC1245 sgRNA- and shRNA-mediated loss-of-function PDAC cells treated with Dox for 48 h. Quantification from three independent experiments is presented as mean ± s.e.m. below; left, ***P = 0.0001 and ****P < 0.0001, as determined by one-way analysis of variance (ANOVA); right, *P = 0.0439 and **P = 0.0024, as determined by two-way ANOVA; NS, not significant. **b**, Cell viability measurements of FC1245 sgCtrl or sgEnpp2 PDAC cells at 72 h; RLU, relative light units. Data indicate mean ± s.e.m. from three experiments; ****P < 0.0001, as determined by one-way ANOVA. **c**, Cell viability measurements of FC1245 PDAC cells treated with vehicle (DMSO) or ATX inhibitor (HA130; 10 μM) for 72 h. Data are shown as mean ± s.e.m. from three experiments; ****P < 0.0001, as determined by two-tailed unpaired *t*-test. **d**, Immunohistochemical staining and quantification of ENPP2 in FC1245 sgRNA- and shRNA-mediated knockdown tumors. Data are represented as mean ± s.e.m. (*n* = 3 mice per arm); **P = 0.0026 and *P = 0.0293, as determined by two-tailed unpaired *t*-test. **e**,**f**, Representative images of FC1245 Dox-inducible *Enpp2-*knockdown tumors (**e**) and tumor weight (**f**) at the experimental end point (–Dox: *n* = 3 mice per arm; +Dox *n* = 5 mice per arm). Data are presented as mean ± s.e.m.; **P = 0.0011, as determined by two-tailed unpaired *t*-test. **g**, Tumor area of the indicated FC1245 *Enpp2*-knockdown tumors measured by ultrasound (–Dox: *n* = 4 mice per arm; +Dox *n* = 5 mice per arm). Data are presented as mean ± s.e.m.; **P = 0.0085 and ***P = 0.0005, as determined by one-way ANOVA. **h**, Immunohistochemical staining and quantification of phospho-AKT (p-AKT; Ser 473) in FC1245 sgCtrl (*n* = 12) and sgEnpp2 (*n* = 11) tumors. Error bars represent mean ± s.e.m.; *P = 0.0148, as determined by two-tailed unpaired *t*-test. **i**, Western blot of FC1245 sgCtrl, sgEnpp2 and reconstituted Enpp2 rescue cell lysates, with quantification presented as mean ± s.e.m. on the right (*n* = 3 independent experiments); left, **P = 0.042; right, **P < 0.0017. Data were analyzed by one-way ANOVA. **j**, Tumor images and tumor weights at the humane end point of sgCtrl (*n* = 3 mice), sgEnpp2 (*n* = 5 mice) and Enpp2 (*n* = 5 mice) rescue tumors. Data are shown as mean ± s.e.m.; *P = 0.0151, **P < 0.0030 and ****P < 0.0001, as determined by one-way ANOVA. Source data

**Extended Data Fig. 1:**
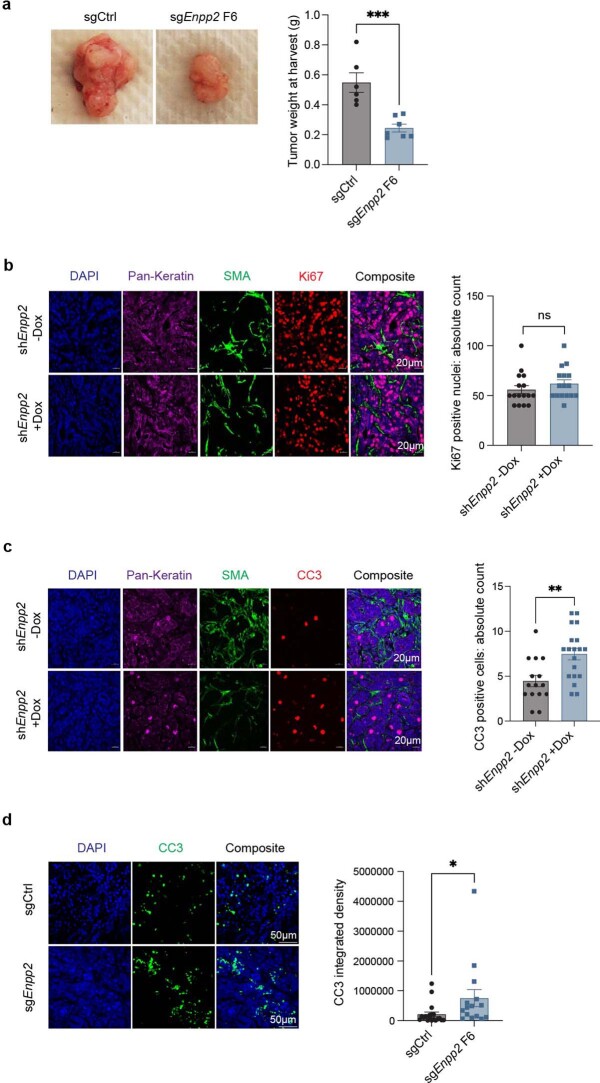
Autotaxin promotes pancreatic tumor progression and suppresses apoptosis in established tumors. **a**, Representative tumor images and weights at humane endpoint of FC1245 sgCtrl (n=6) and sg*Enpp2* (n=7) tumors. Data are presented as mean ± SEM. ***P= 0.0008 by two-tailed unpaired t-test. **b**, Immunohistochemical staining and quantification of Ki-67 and the indicated markers in FC1245 Dox inducible control and *Enpp2* knockdown tumors. Scale bar = 20 mm. Data are shown as mean ± SEM (n = 16 ROIs from 4 mice per group). *ns* = not significant by two-tailed unpaired t-test. **c**, Immunohistochemical staining and quantification of cleaved caspase-3 in Dox inducible control and *Enpp2* knockdown tumors. Scale bar = 20 mm. Data are plotted as mean ± SEM (-Dox: n = 15 ROIs, +Dox: n = 19 ROIs from 4 mice per group). **P = 0.0027 by two-tailed unpaired t-test. **d**, Immunohistochemical staining and quantification of cleaved caspase-3 in FC1245 sgCtrl and sg*Enpp2* tumors. Scale bar = 50 mm. Data are plotted as mean ± SEM (sgCtrl: n = 19 ROIs, sg*Enpp2*: n = 15 ROIs from 4 mice per group). *P = 0.0255 by two-tailed unpaired t-test. Source data

To analyze the significance of lysolipid signaling in the regulation of the PDAC microenvironment, we developed ATX loss-of-function systems in PDAC cells amenable to orthotopic implantation in immune-competent hosts. These included constitutive knockdown lines of *Enpp2* (encoding ATX) generated by transient transfection of Cas9 and single guide *Enpp2* (sgEnpp2) sequences as well as doxycycline (Dox)-inducible short hairpin RNAs (shRNAs) targeting *Enpp2* to enable genetic ATX inhibition in established tumors. Both approaches successfully reduced ATX expression at the protein level (Fig. 1a). Proliferation rates were significantly, but only modestly, reduced by ATX genetic inhibition (Fig. 1b), similar to proliferation rates observed after treatment of PDAC cells with the ATX inhibitor HA130 (Fig. 1c). After implantation into the pancreas, constitutive ATX inhibition reduced PDAC growth as expected (Extended Data Fig. 1a). Reduction of ATX protein expression using both constitutive and inducible knockdown approaches was successfully validated on orthotopically transplanted tumor sections (Fig. 1d). Inducible ATX inhibition in established PDAC also reduced tumor growth (Fig. 1e–g) and reduced AKT activation in vivo (Fig. 1h), consistent with previous findings^24^. However, after ATX extinction, proliferation in vivo was not altered, unlike the effects observed with constitutive ATX inhibition^24^ (Extended Data Fig. 1b). Apoptosis increased in the *Enpp2*-knockdown tumors using either loss-of-function approach (Extended Data Fig. 1c,d), indicating a role for cancer cell-derived ATX in PDAC progression, which suppresses tumor cell death. Rescuing ATX expression in constitutive *Enpp2*-knockdown lines partially rescued tumor growth after implantation into the pancreas, underlining the tumor growth-supportive function of ATX (Fig. 1i,j).

### ATX signaling limits intratumor eosinophil abundance

**Fig. 2:**
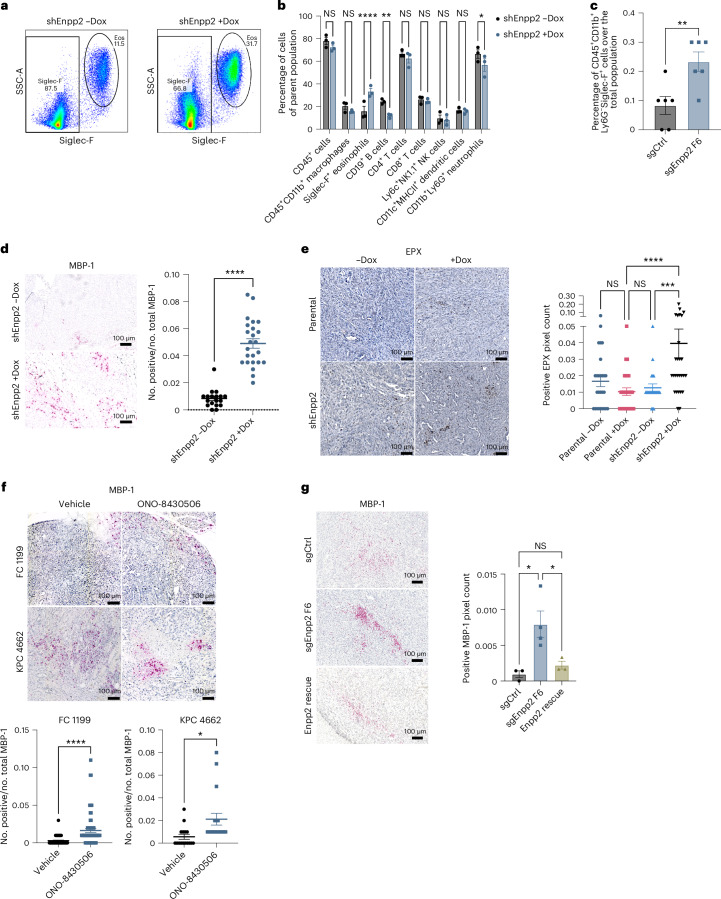
ATX signaling limits intratumor eosinophil abundance. **a**, Representative flow cytometry density plots of CD45^+^CD11b^+^CD64^lo^Ly6G^−^CD11c^lo^ cells, indicating Siglec-F^+^ eosinophil abundance in a Dox-inducible *Enpp2*-knockdown model (*n* = 3 mice per group); Eos, eosinophil. **b**, Quantification of flow cytometry-based immune profiling of Dox-inducible *Enpp2*-knockdown tumors. Data are presented as mean ± s.e.m. (*n* = 3 mice per condition); *P = 0.0192, **P = 0.0032 and ****P < 0.0001, as determined by two-way ANOVA; NK, natural killer; MHCII, major histocompatibility complex class II. **c**, Relative frequency of CD45^+^CD11b^+^Ly6G^−^ cells indicating Siglec-F^+^ eosinophil abundance in sgCtrl and sgEnpp2 FC1245 tumors. Data are shown as mean ± s.e.m. (*n* = 6 mice per group); **P = 0.0079, as determined by two-tailed unpaired *t*-test. **d**, Immunohistochemical staining and quantification of MBP-1 in FC1245 control (*n* = 9 samples) and Dox-inducible sgEnpp2 (*n* = 10 samples) tumors. Data are shown as mean ± s.e.m. (*n* = <5 fields per tumor); ****P < 0.0001, as determined by two-tailed unpaired *t*-test. **e**, Immunohistochemical staining and quantification of EPX in FC1245 control and Dox-inducible sgEnpp2 tumors. Data are presented as mean ± s.e.m. (*n* = 9 tumors per group); ***P = 0.0005 and ****P < 0.0001, as determined by one-way ANOVA. **f**, MBP-1 immunohistochemical staining and quantification of tumors from two PDAC models treated with vehicle or ATX inhibitor ONO-8430506 (30 mg per kg (body weight)). Data are shown as mean ± s.e.m., with *n* = 5 tumors per group for the 4662 model and *n* = 9 tumors per group for the FC1199 model; *P = 0.0129 and ****P < 0.0001, as determined by two-tailed unpaired *t*-test. **g**, Immunohistochemical staining and quantification of MBP-1 in FC1245 sgCtrl, sgEnpp2 and ENPP2 rescue tumors. Data are shown as mean ± s.e.m. (*n* = 3 tumors per group); from left to right, *P = 0.0203 and *P = 0.0486, as determined by two-tailed unpaired *t*-test. Source data

**Extended Data Fig. 2:**
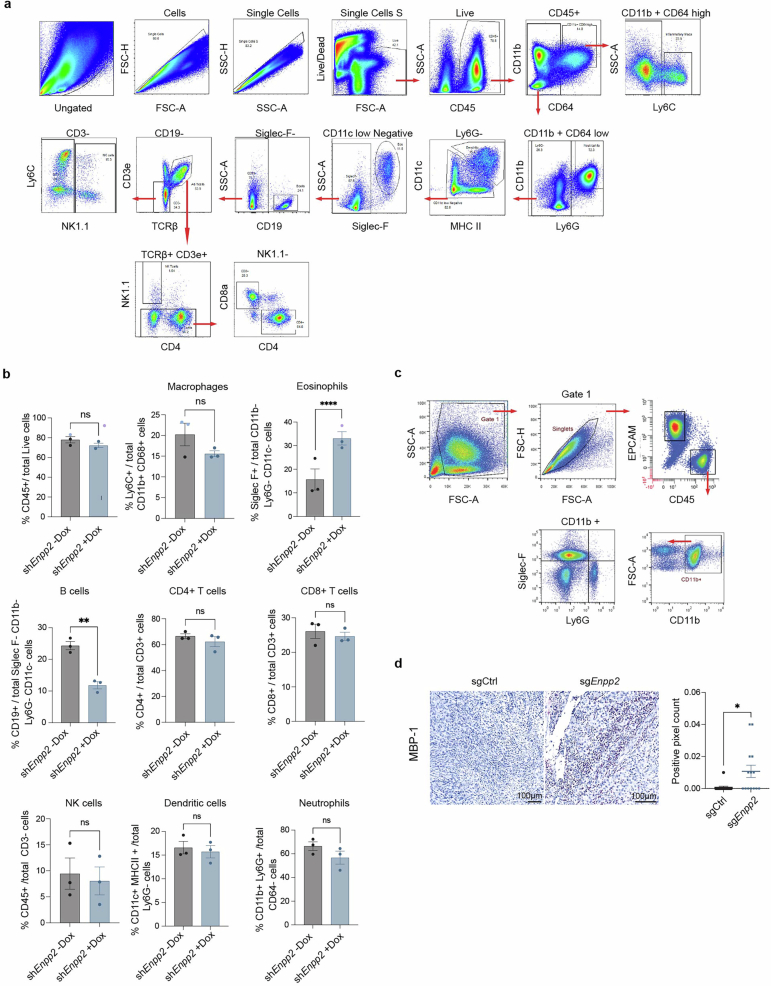
Autotaxin-LPA signaling regulates pancreatic cancer immune contexture including suppression of eosinophil infiltration. **a**, Spectral flow cytometry gating strategy used for isolation of pure FACS sorted eosinophil populations from Dox inducible control and sh*Enpp2* tumors. **b**, Quantification of the flow cytometry-based immune profiling of Dox inducible *Enpp2* knockdown tumors. Data are presented as mean ± SEM (n = 3 tumors per group). *ns*= not significant, **P= 0.0032, ****P< 0.0001 by two-way ANOVA. **c**, Flow cytometry gating strategy used for isolation of eosinophil populations from FC1245 sgCtrl and sg*Enpp2* tumors. **d**, Immunohistochemical staining and quantitation of MBP-1 in FC1245 sgCtrl and sg*Enpp2* tumors. Scale bar = 100 mm. Data are shown as mean ± SEM (n = 14 ROIs from 4 tumors per group). *P= 0.0168 by two-tailed unpaired t-test. Source data

Given the potential impact of the ATX–LPA axis on tumor immune contexture^14,18^, we analyzed the regulation of the PDAC immune microenvironment by ATX signaling. After confirming PDAC formation by high-resolution ultrasound, we administered Dox to mice harboring Dox-inducible shControl (shCtrl)- or shEnpp2-expressing tumors and collected tumors for immune phenotyping by spectral flow cytometry (Fig. 2a and Extended Data Fig. 2a). Although ATX–LPA signaling suppresses CD8^+^ T cell infiltration into tumors in other tissue contexts, T cell frequencies were not impacted after ATX inhibition. However, several immune cell subsets were regulated by ATX–LPA, with the most marked change among eosinophils (Fig. 2b and Extended Data Fig. 2b). Eosinophil abundance increased nearly threefold after inhibition of ATX, raising the possibility that ATX–LPA signaling suppresses eosinophil accumulation in the PDAC microenvironment. Although mechanisms and consequences of eosinophil recruitment into pancreatic tumors have not been investigated, low circulating eosinophil frequencies and low eosinophil-to-lymphocyte ratios were found to be significant independent prognostic factors of poor outcomes among individuals with PDAC^25^, prompting us to further investigate the link between ATX–LPA signaling and eosinophils. We established tumors using an independent ATX loss-of-function system, performed flow cytometry and confirmed an increase in intratumor eosinophils with ATX inhibition (Fig. 2c and Extended Data Fig. 2c). To validate these findings in intact tumor tissues, we quantified eosinophil abundance by immunohistochemical staining for the eosinophil markers major basic protein (MBP) and eosinophil peroxidase (EPX). Analysis of both markers confirmed increased eosinophil abundance with ATX inhibition across loss-of-function systems (Fig. 2d,e and Extended Data Fig. 2d). We next treated PDAC-bearing mice with the pharmacologic ATX inhibitor ONO-8430506 (ref. ^26^), which reduced PDAC growth in a previous study^24^. Pharmacologic ATX inhibition increased eosinophil abundance in PDAC (Fig. 2f), further supporting a role for ATX in the suppression of eosinophil accumulation in the pancreatic tumor microenvironment. Notably, restoring ATX expression in constitutive *Enpp2*-knockdown PDAC cells significantly reduced tumor-infiltrating eosinophil abundance and restored eosinophil numbers to baseline levels after implantation into the pancreas (Fig. 2g), highlighting the negative regulatory effect of ATX on tumor eosinophil abundance. We were motivated to investigate the significance of eosinophil infiltration in this context in light of recent evidence of the antitumor potential of tumor-infiltrating eosinophils in colorectal cancer^27^, hepatocellular carcinoma and breast cancer^28,29^ and emerging evidence linking intratumor eosinophils to tumor growth suppression via interactions with tumor cells and lymphocytes^30^.

### Intratumor eosinophils infiltrate human and mouse PDAC tissue

**Fig. 3:**
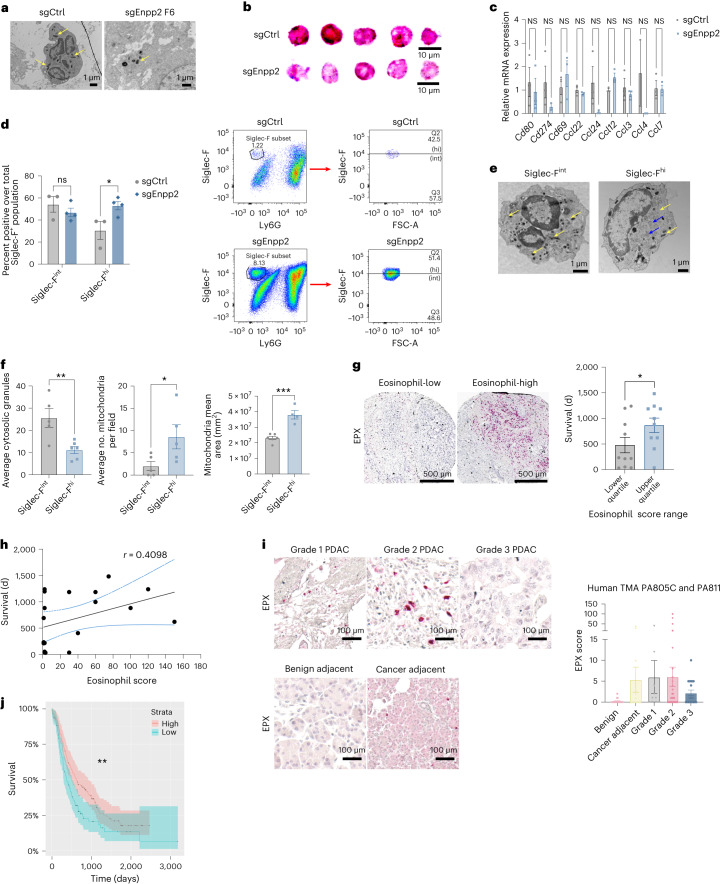
Intratumor eosinophils infiltrate human and mouse PDAC tissue. **a**, Representative TEM images depicting eosinophils derived from sgCtrl and sgEnpp2 FC1245 tumors (*n* = 3 tumors per group). Arrows denote cytosolic granules. **b**, Wright’s Giemsa histological staining of eosinophil populations pooled from sgCtrl or sgEnpp2 tumors and isolated by FACS (*n* = 3 tumors per group). **c**, Real-time quantitative PCR (RT–qPCR) transcript profiling of FACS-isolated eosinophils from sgCtrl and sgEnpp2 FC1245 tumors (*n* = 3 tumors per arm plotted as mean ± s.e.m.); data were analyzed by two-way ANOVA. **d**, Percentage of Siglec-F^int^ and Siglec-F^hi^ eosinophil abundance in sgCtrl (*n* = 3 mice) and sgEnpp2 (*n* = 4 mice) FC1245 tumors over the total Siglec-F^+^ population. Data are presented as mean ± s.e.m.; *P = 0.0331, as determined by two-way ANOVA. **e**, TEM of sorted eosinophils from established PDAC representing both intermediate and high Siglec-F expression (*n* = 3 tumors). Arrowheads denote cytosolic granules (yellow) and mitochondria (blue). **f**, Cytosolic granules (intermediate: *n* = 5 samples; high: *n* = 6 samples) and mitochondria (*n* = 5 samples) counts of eosinophil populations isolated from PDAC tumors expressing intermediate and high levels of Siglec-F. Data are plotted as mean ± s.e.m.; *P = 0.0270, **P = 0.0075 and ***P = 0.0004, as determined by two-tailed unpaired *t*-test. **g**, Left, representative EPX staining of human PDAC from a tumor microarray depicting eosinophil-low and eosinophil-high samples. Right, overall survival among individuals within the lower and upper quartiles of eosinophil abundance; the analysis was limited to primary tumor samples on the array with known survival outcomes. Data are presented as mean ± s.e.m. (*n* = 10 mice per quartile); *P = 0.0390, as determined by one-tailed unpaired *t*-test. **h**, Simple linear regression correlation plot of eosinophil abundance with overall survival from the tumor microarray depicted in **g**. Data are presented as mean ± 95% confidence interval (95% CI). **i**, Representative images and quantification of EPX staining from two independent PDAC tumor microarrays across tumor grades. Data are presented as mean ± s.e.m. **j**, Kaplan–Meier analysis of individuals with low (*n* = 93 samples) versus high (*n* = 121 samples) expression of *EPX*; **P = 0.0085, as determined by log-rank test. Source data

As PDAC intratumor eosinophils have been reported in few studies^31,32^, we aimed to confirm eosinophil identities in mouse and human PDAC and assess their functional significance. We established tumors in mice and collected tumor tissue for transmission electron microscopy (TEM) analysis of intact tissue. TEM analysis revealed characteristic eosinophil structures, including sombrero vesicles and granules. Notably, eosinophil ultrastructure differed noticeably between genotypes, with evidence of increased degranulation consistent with increased eosinophil activation in the ATX-inhibited context and evidence of ring-like bilobed nuclei in control tumors, as previously described for resolving or homeostatic eosinophils^33^ (Fig. 3a), suggesting regulation of both eosinophil abundance and phenotype by cancer cell-derived ATX. To further investigate the regulation of eosinophil phenotypes by cancer cell-derived ATX, we sorted eosinophils based on the cell-surface markers used for flow cytometry analysis (Extended Data Fig. 2c) and assessed cellular morphology by Wright’s Giemsa differential staining. Analysis of differentially stained, pure sorted eosinophil populations from ATX-replete and ATX-depleted tumors revealed differences in morphological features across the genotypes. Eosinophils from *Enpp2*-knockdown tumors displayed irregularly organized membrane architecture, multilobed segmented nuclei, empty vacuoles within the cytoplasm and less intense staining of cytosolic granules (Fig. 3b), potentially indicating eosinophil activation and degranulation in the *Enpp2*-knockdown context, consistent with our tumor tissue TEM analysis. To further characterize functional differences across eosinophil populations from ATX-replete and constitutive ATX-depleted tumors, we isolated mRNA from sorted eosinophil populations and analyzed gene expression, including eosinophil cell-surface markers and chemokines (Fig. 3c). Eosinophils from *Enpp2*-knockdown tumors trended toward reduced expression of *Cd274*, *Ccl4* and *Ccl24*, but gene expression was highly variable, and these differences did not reach statistical significance. During our flow cytometry analysis, we noted a range of Siglec-F levels on intratumor eosinophils. Eosinophil heterogeneity with respect to Siglec-F expression bears relevance in light of recent work implicating Siglec-F^hi^ eosinophils in inflammatory processes and Siglec-F^int^ eosinophils in the negative regulation of immune responses and tissue homeostasis^33^. We found that Siglec-F^hi^ eosinophils were specifically elevated in the ATX-depleted setting as a proportion of total eosinophils (Fig. 3d). To assess the significance of these observed differences in Siglec-F surface levels, we isolated Siglec-F^hi^ and Siglec-F^int^ eosinophils from PDAC and subjected them to TEM analysis. TEM analysis revealed ultrastructural differences between Siglec-F^hi^ and Siglec-F^int^ eosinophils, with characteristic oblong granules with rectangular crystals among Siglec-F^hi^ cells and round granules with an osmophilic core among Siglec-F^int^ cells. Quantitative analysis of morphological presentations across Siglec-F^hi^ and Siglec-F^int^ eosinophils revealed significant differences in storage granule number (significantly more in Siglec-F^int^ eosinophils), number of mitochondria (significantly more in Siglec-F^hi^ eosinophils) and mitochondrial area (significantly more in Siglec-F^hi^ eosinophils; Fig. 3e,f). Together, our phenotypic and functional characterization of eosinophil populations reveal that cross-talk between PDAC cell-derived ATX and eosinophils results in phenotypic alterations associated with eosinophil activation.

To query the presence of eosinophils in human PDAC, we stained three tumor microarrays containing surgically resected specimens from independent populations of individuals with PDAC for the eosinophil marker EPX. These arrays revealed the presence of eosinophils in human PDAC tissues, with considerable heterogeneity in abundance (Fig. 3g). As outcomes were known for one of these tumor microarrays, we assessed the prognostic significance of eosinophil frequencies and found that rare individuals with the highest eosinophil abundance had the best prognosis with respect to overall survival (Fig. 3g) and that eosinophil abundance and survival time were positively correlated (Fig. 3h), although we note that these associations were modest. As tumor grade data were available for two of these tumor microarrays, we analyzed tumor eosinophil infiltration across different PDAC grades. Across the two different tumor microarrays, eosinophil abundance trended toward reduced abundance in more advanced grade 3 compared to grade 1 and grade 2 tumors, although again we note that the association between eosinophil abundance and tumor grade was modest. Overall, eosinophils were also more abundant in cancer-adjacent pancreatic tissue than in benign tissue cores (Fig. 3i). To further analyze the prognostic significance of intratumor eosinophils, we queried the RNA-sequencing profiles of individuals with PDAC with known associated outcomes and found that individuals with higher *EPX* expression had a slightly better prognosis than those with low *EPX* expression (Fig. 3j). Together, these results indicate that mouse and human PDAC harbor intratumor eosinophils and that these eosinophils within the tumor microenvironment associate with better prognosis among individuals with PDAC.

### ATX signaling curbs eosinophil tumor-restraining potential

**Fig. 4:**
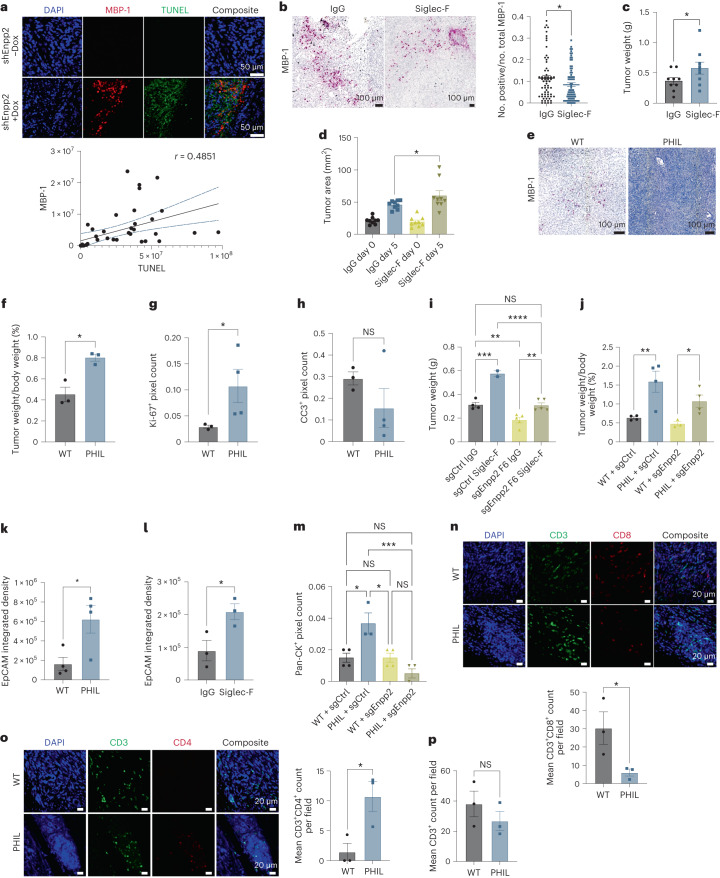
ATX signaling curbs eosinophil tumor-restraining potential. **a**, MBP-1 and TUNEL staining in FC1245 tumors with a correlation (*n* = 9 tumors), plotted as mean ± 95% CI. **b**, MBP-1 staining of FC1245 tumors, presented as mean ± s.e.m. (IgG: 62 regions of interest (ROIs) from *n* = 8 tumors; Siglec-F: 76 ROIs from *n* = 8 tumors); *P = 0.0477, as determined by two-tailed unpaired *t*-test. **c**, FC1245 tumor weights (IgG: *n* = 9; Siglec-F: *n* = 8), plotted as mean ± s.e.m.; *P = 0.0402, as determined by two-tailed unpaired *t*-test. **d**, Ultrasound measurements from **c**, plotted as mean ± s.e.m.; *P = 0.0464, as determined by two-tailed unpaired *t*-test. **e**, MBP-1 staining (*n* = 3 tumors). **f**, Tumor-to-body weight ratio, plotted as mean ± s.e.m. (*n* = 3 tumors); *P = 0.0103 by two-tailed unpaired *t*-test. **g**, Ki-67 quantification, presented as mean ± s.e.m. (WT: *n* = 3 tumors; PHIL: *n* = 4 tumors); *P = 0.0477, as determined by one-tailed unpaired *t*-test. **h**, Cleaved caspase-3 (CC3) quantification, plotted as mean ± s.e.m. (WT: *n* = 3 tumors; PHIL: *n* = 4 tumors); data were analyzed by two-tailed unpaired *t*-test. **i**, Tumor weights of FC1245 sgCtrl (IgG: *n* = 4 mice; Siglec-F: *n* = 2 mice) and sgEnpp2 (IgG: *n* = 5 mice; Siglec-F: *n* = 5 mice) mice, plotted as mean ± s.e.m.; **P = 0.0058, **P = 0.0069, ***P = 0.0002 and ****P < 0.0001, as determined by one-way ANOVA. **j**, Tumor-to-body weight ratio of WT or PHIL mice harboring FC1245 sgCtrl or sgEnpp2 tumors, presented as mean ± s.e.m. (*n* = 4 tumors); *P = 0.0441 and **P = 0.0023, as determined by one-way ANOVA. **k**, Quantification of liver metastatic nodules. Data are shown as mean ± s.e.m. (*n* = 3 tumors per arm); *P = 0.0268, as determined by two-tailed unpaired *t*-test. **l**, Quantification of liver metastasis in FC1245 mice treated with IgG or Siglec-F, plotted as mean ± s.e.m. (*n* = 3 tumors per arm); *P = 0.0400, as determined by two-tailed unpaired *t*-test. **m**, Quantification of liver metastatic nodules in WT or PHIL mice harboring FC1245 sgCtrl or sgEnpp2 tumors, plotted as mean ± s.e.m. (*n* = 4 tumors per condition); *P = 0.0104 and ***P = 0.0006, as determined by one-way ANOVA. **n**, CD3 and CD8 staining, plotted as mean ± s.e.m. (*n* = 3 tumors per group); *P = 0.0281, as determined by two-tailed unpaired *t*-test. **o**, CD3 and CD4 staining, plotted as mean ± s.e.m. (*n* = 3 tumors per arm); *P = 0.0334, as determined by two-tailed unpaired *t*-test. **p**, CD3^+^ counts, plotted as mean ± s.e.m. (*n* = 3 mice per arm); data were analyzed by two-tailed unpaired *t*-test. Source data

**Extended Data Fig. 3:**
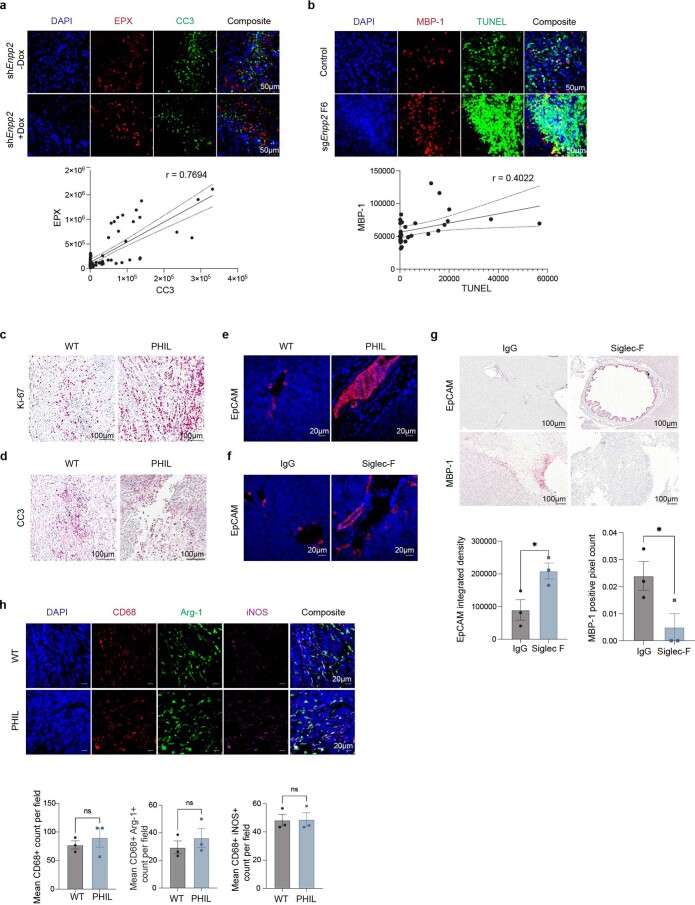
Eosinophils underlie the anti-tumor response to autotaxin inhibition. **a**, Representative immunohistochemical staining for EPX and CC3 in control (n = 9 mice) and dox inducible (n = 10 mice) *Enpp2* knockdown tumors. Correlation analysis representative of ≤5 fields per tumor plotted below. Data are plotted as mean ± 95% CI. Scale bar = 50 mm. **b**, Immunohistochemical staining and quantitation of MBP-1 and TUNEL in FC1245 sgCtrl (n=9 samples) and sg*Enpp2* (n=10 samples) tumors. Correlation analysis plotted below from at least 5 fields per tumor and represented as mean ± 95% CI. Scale bar = 50 mm. **c**, Representative immunohistological staining of Ki-67 positive nuclei in wild type and PHIL tumors. Scale bar = 100 mm (n = 3 tumors per group). **d**, Representative immunohistological staining of cleaved caspase-3 in wild type and PHIL tumors. Scale bar = 100 mm (n = 3 tumors per group). **e**, Representative immunohistological staining of EpCAM positive liver metastatic nodules in wild type and PHIL tumors. Scale bar = 100 mm (n = 3 tumors per group). **f**, Representative immunohistological staining of EpCAM positive liver metastatic nodules in IgG versus Siglec F treated FC1245 PDAC liver metastatic nodules. Scale bar = 100 mm (n = 3 tumors per group). **g**, Representative immunohistological staining and quantification of EpCAM in IgG versus Siglec F treated FC1245 PDAC liver metastatic nodules, and representative immunohistological staining and quantification of MBP-1 in IgG versus Siglec-F treated FC1245 PDAC tumors to validate eosinophil depletion upon Siglec F administration. Data are presented as mean ± SEM. Scale bar = 100 mm (n = 3 tumors per group). Left: *P = 0.0040; right: *P = 0.0297 by two-tailed unpaired t-test. **h**, Immunohistological staining and quantification of macrophage subpopulations in wild-type and PHIL mice. Scale bar = 20 mm. Data are shown as mean ± SEM (n=3 mice per group). *ns*= not significant by two-tailed unpaired t-test. Source data

We next addressed the functional significance of eosinophils in PDAC tissues. In diverse disease states, inflammatory mediators, such as cytokines and chemokines, recruit eosinophils to affected sites, where they facilitate the response to infection and tissue damage^34^. Classical eosinophil functions in the context of allergic diseases or host infection with microbial pathogens, including production of lipid mediators and cationic proteins (EPX, MBP and eosinophil cationic protein), may be pro- or antitumorigenic depending on tissue setting^35,36^. As the characteristic eosinophil granules store cytotoxic mediators (that is, EPX and MBP) released at sites of inflammation^37^, we initially assessed an association between eosinophils and apoptosis across the PDAC microenvironment. To this end, we stained for eosinophil markers EPX and MBP and for cleaved caspase-3 and TUNEL to assay apoptosis and analyzed spatial relationships between markers for eosinophils and apoptosis. We found a significant correlation between eosinophil abundance and apoptosis in *Enpp2*-knockdown PDAC tissues using different markers for eosinophils and apoptosis and two independent loss-of-function systems (Fig. 4a and Extended Data Fig. 3a,b), suggesting antitumor potential. To probe the significance of eosinophils in PDAC progression, we performed eosinophil depletion experiments using two independent approaches. In our first approach, we treated PDAC-bearing mice with IgG control antibody or with depleting antibody targeting Siglec-F^38^. This intervention reduced intratumor eosinophils, as expected, and modestly, albeit significantly, increased tumor growth (Fig. 4b–d). As an orthogonal approach, we analyzed PDAC progression in PHIL mice, a transgenic strain that lacks eosinophils because of diphtheria toxin expression driven by the *Epx* promoter^39^. Compared to tumors in littermate controls, PDAC in PHIL mice harbored fewer eosinophils, as expected (Fig. 4e), and exhibited modestly increased growth (Fig. 4f), in this case normalized to body weight as PHIL mice are smaller than their control littermates. We analyzed changes in cell proliferation across wild-type (WT) and PHIL tumors and we observed a significant increase in Ki-67^+^ nuclei in eosinophil-deficient tumors (Fig. 4g and Extended Data Fig. 3c). Staining for cleaved caspase-3 (a marker of apoptosis) showed no significant changes in apoptosis between WT and PHIL tumors (Fig. 4h and Extended Data Fig. 3d). We reasoned that these modest impacts on tumor progression after eosinophil depletion may be due to the small number of eosinophils in control tumors and that eosinophil infiltration underlies, at least in part, the antitumor response to ATX inhibition. To test this possibility, we inhibited eosinophils with Siglec-F-depleting antibody and used PHIL mice to analyze the progression of control or ATX-deficient PDAC. Both models of eosinophil deficiency restored the growth of ATX-null PDAC to control levels (Fig. 4i,j), implicating the suppression of eosinophil accumulation as a tumor-promoting mechanism of ATX–LPA signaling. We note that the difference in tumor growth between eosinophil-replete control and sgEnpp2 arms was muted in the experiments in PHIL mice (Fig. 4j), as these experiments were terminated sooner than experiments in only WT mice due to disease severity in the PHIL hosts. Using Siglec-F-depleting antibody to deplete eosinophils from established tumors and in PHIL mice, we also noticed that eosinophil deficiency enabled metastatic spread of a PDAC model with otherwise limited metastatic ability, implicating eosinophils as negative regulators of metastasis (Fig. 4k,l and Extended Data Fig. 3e–g); this prometastatic consequence of eosinophil deficiency was lost in the absence of cancer cell-derived ATX (Fig. 4m), consistent with a reciprocal interaction between eosinophils and ATX in the regulation of tumor progression.

We further investigated immune microenvironment changes between PHIL mice and WT control littermates, and our findings revealed a significant decrease in the abundance of cytotoxic CD8^+^ T cell populations in PHIL tumors (Fig. 4n). Conversely, we found a significant increase in CD4^+^ T cell populations in PHIL tumors, whereas the overall abundance of the CD3^+^ pan-T cell marker remained comparable across genotypes (Fig. 4o,p). Our analysis of macrophage subpopulations across tumors derived from PHIL mice and WT control littermates revealed no significant changes in the distribution of the pan-macrophage marker CD68 nor in macrophages positive for ARG1 or inducible nitric oxide synthase (Extended Data Fig. 3h). Together, these results suggest potential regulation of PDAC progression and CD8^+^ T cell infiltration by eosinophils.

### ATX disrupts AP-1 activity and *Ccl11* expression

**Fig. 5:**
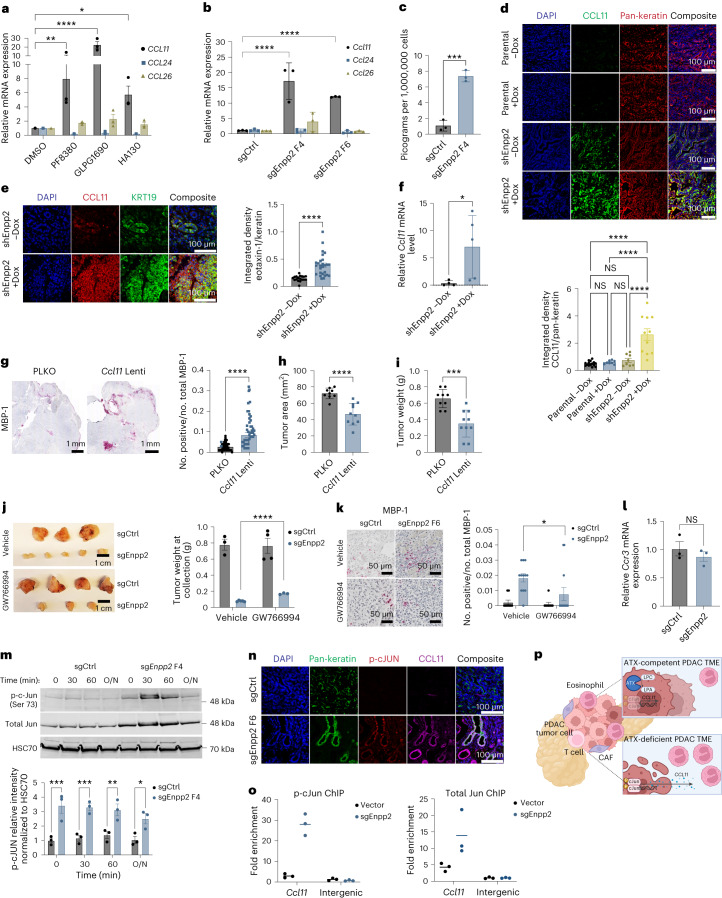
ATX disrupts AP-1 activity and *Ccl11* expression. **a**, Transcript levels in 8988T human PDAC cells treated with the indicated ATX inhibitors at a concentration of 10 μM for 24 h. Data are presented as mean ± s.e.m. from three experiments; *P = 0.0335, **P = 0.0029 and ****P < 0.0001, as determined by two-way ANOVA. **b**, Quantification of the indicated transcript targets in FC1245 cells, plotted as mean ± s.e.m. (*n* = 3 experiments); ****P < 0.0001, as determined by two-way ANOVA. **c**, CCL11 concentration in conditioned medium, shown as mean ± s.e.m. from three experiments; ***P = 0.0004, as determined by two-tailed unpaired *t*-test. **d**, Immunohistochemistry data plotted as mean ± s.e.m. (parental –Dox: *n* = 31 ROIs from three tumors; parental +Dox: *n* = 24 ROIs from three tumors; shEnpp2 –Dox: *n* = 16 ROIs from two tumors; shEnpp2 +Dox: *n* = 18 ROIs from three tumors); ****P < 0.0001, as determined by one-way ANOVA. **e**, RNA fluorescence in situ hybridization data, presented as mean ± s.e.m. (–Dox: *n* = 18 ROIs from two mice; +Dox: *n* = 27 ROIs from three mice); ****P < 0.0001, as determined by two-tailed unpaired *t*-test. **f**, *Ccl11* mRNA expression from EpCAM^+^ tumor cells, presented as mean ± s.e.m. (*n* = 5 tumors per group); *P = 0.0317, as determined by two-tailed unpaired *t*-test. **g**, MBP-1 staining in vector (*n* = 45 ROIs from five FC1245 tumors) or *Ccl11*-overexpressing (*n* = 45 ROIs from nine FC1245 tumors) tumors. Data are shown as mean ± s.e.m.; ****P < 0.0001, as assessed by two-tailed unpaired *t*-test; Lenti, lentivirus; PLKO, empty lentiviral vector. **h**,**i**, Tumor size, as determined by ultrasound (**h**) and tumor weights (**i**), presented as mean ± s.e.m. (*n* = 9 per group); ***P = 0.0002 and ****P < 0.0001, as determined by two-tailed unpaired *t*-test. **j**, Tumor images and weights, plotted as mean ± s.e.m. (*n* = 3–4 mice per condition); ****P < 0.0001, as determined by two-way ANOVA. **k**, MBP-1 staining, plotted as mean ± s.e.m. (sgCtrl: *n* = 9 ROIs; sgEnpp2: *n* = 13 ROIs from *n* = 3 mice per group); *P = 0.0500, as determined by two-tailed unpaired *t*-test. **l**, *Ccr3* mRNA from sorted eosinophils, presented as mean ± s.e.m. (*n* = 3 tumors per group); data were analyzed by two-tailed unpaired *t*-test. **m**, Western blots of FC1245 nuclear lysates with quantification plotted as mean ± s.e.m. (*n* = 3 independent experiments); *P = 0.0212, **P = 0.0059, ***P = 0.0009 (0 min) and ***P = 0.0003 (30 min), as determined by two-way ANOVA; O/N, overnight; p-c-Jun, phospho-c-Jun. **n**, Staining of FC1245 tumors (*n* = 4 mice per group). **o**, Chromatin immunoprecipitation (ChIP) of FC1245 cells followed by RT–qPCR for the *Ccl11* promoter, plotted as individual technical triplicates with means. **p**, Schematic depicting interactions among ATX signaling, eotaxin regulation and eosinophil infiltration in PDAC; CAF, cancer-associated fibroblast; LPC, lysophosphatidylcholine; TME, tumor microenvironment. Source data

**Extended Data Fig. 4:**
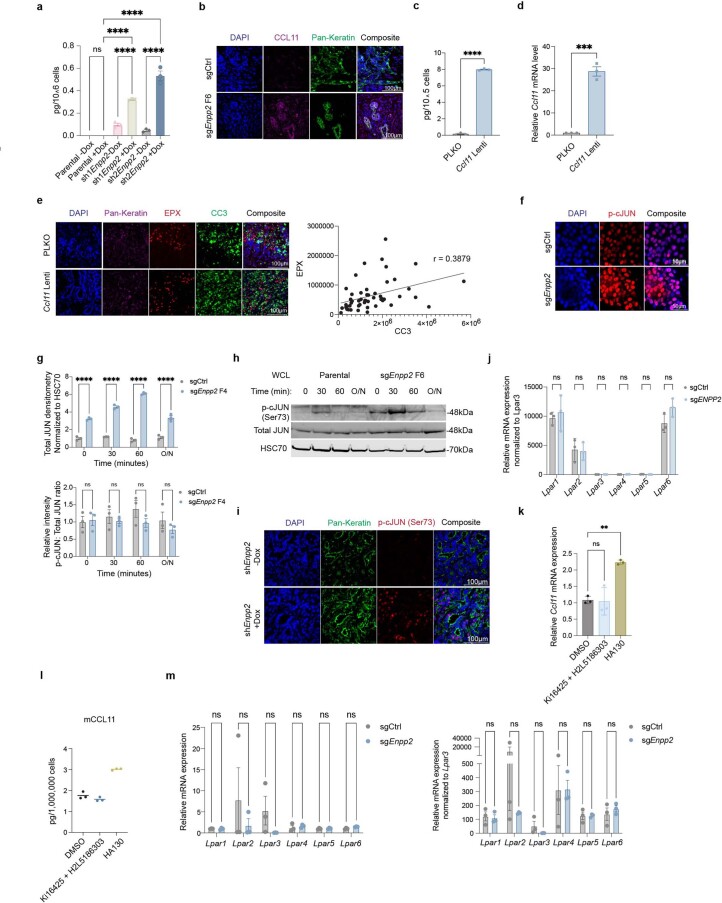
Autotaxin signaling negatively regulates c-Jun and expression of *Ccl11* in tumor cells. **a**, CCL11 ELISA of FC1245 control and *Enpp2* knockdown cells, plotted as mean ± SEM from 3 experiments. ****P< 0.0001 by one-way ANOVA. **b**, CCL11 and pan-Keratin staining in FC1245 sgCtrl and sg*Enpp2* tumors (n = 4 samples per group). Scale bar = 100 mm. **c**, CCL11 ELISA of control or CCL11-overexpressing FC1245 cells, plotted as mean ± SEM from 3 experiments. ****P< 0.0001 by two-tailed unpaired t-test. **d**, *Ccl11* transcript level in the indicated cell lines, presented as mean ± SEM from 3 experiments. ***P= 0.0002 by two-tailed unpaired t-test. **e**, Immunohistochemical staining for the indicated markers of control or CCL11-expressing tumors (n = 51 ROIs from 7 mice per group) with correlation shown on the right. Scale bar = 100mm. **f**, Representative p-cJUN staining in FC1245 sgCtrl and sg*Enpp2* cells from 3 experiments. Scale bar = 50mm. **g**, Densitometry of total JUN and p-cJUN/total JUN ratio of blots from Fig. (5l). Data presented as mean ± SEM from 3 independent experiments. *ns* = not significant, ****P< 0.0001 by two-way ANOVA. **h**, p-cJUN and total JUN protein abundance in FC1245 sgCtrl or sg*Enpp2* whole cell lysates at the indicated time point. **i**, Immunohistochemical staining for the indicated markers on Dox inducible control or *Enpp2* knockdown tumors, representative of n = 4 tumors per group. Scale bar = 100 mm. **j**, LPAR1-6 transcript level in FC1245 sg*Ctrl* and sg*Enpp2* cells, plotted as mean ± SEM from 3 independent experiments. *ns* = not significant by two-way ANOVA. **k**, *Ccl11* mRNA levels in FC1245 cells treated with 10 mM LPAR inhibitors (Ki16425 + H2L5186303) or ENPP2 inhibitor (HA130) for 24 hours, presented as mean ± SEM from 3 experiments. *ns* = not significant, ****P< 0.0001 by one-way ANOVA. **l**, CCL11 quantikine array of FC1245 cells treated with 10 mM LPAR inhibitors (Ki16425 + H2L5186303) or ENPP2 inhibitor (HA130) for 24 hours. Data represents mean from 3 technical replicates. **m**, qPCR transcript analysis of LPAR1-6 in FACS isolated eosinophils from FC1245 sgCtrl and sg*Enpp2* tumors, plotted as mean ± SEM from 3 independent experiments. *ns* = not significant by two-way ANOVA. Source data

We next investigated the mechanism by which ATX suppresses eosinophil accumulation in the PDAC microenvironment. Eosinophils are recruited into inflamed tissues by eotaxins, including eotaxin-1 (CCL11), eotaxin-2 (CCL24) and eotaxin-3 (CCL26)^40^. We analyzed the expression of these three chemokines in human PDAC cells treated with the pharmacologic ATX inhibitors HA130 (ref. ^41^), PF-8380 (ref. ^42^) and GLPG1690 (ref. ^43^) and found that *CCL11* was uniquely upregulated after the inhibition of ATX (Fig. 5a). Consistent with these results, we found that the expression of *Ccl11* was upregulated in mouse PDAC cells genetically deficient for ATX, whereas the expression levels of *Ccl24* and *Ccl26* were unperturbed (Fig. 5b). As secreted CCL11 protein was below the detection limit in the PDAC cell culture medium, we treated cells with inflammatory mediators tumor necrosis factor-α (TNFα) and interleukin-4 (IL-4). Inflammatory stimuli induced CCL11 secretion by PDAC cells, which was markedly elevated by ATX-deficient PDAC cells across independent clones and loss-of-function systems compared to control cells (Fig. 5c and Extended Data Fig. 4a). To assess this relationship in vivo, we stained for CCL11 in control and ATX-deficient tumors and found greater CCL11 expression when ATX was inhibited, either constitutively or inducibly (Fig. 5d and Extended Data Fig. 4b), which was barely detectable in ATX-expressing tumor cells. As CCL11 is a secreted protein, we confirmed these finding as well as the cancer cell origin of this chemokine by staining for *Ccl11* and *Krt19* using RNA fluorescence in situ hybridization. This analysis revealed greater *Ccl11* expression by PDAC cells deficient in ATX (Fig. 5e). Next, we established control and ATX-deficient tumors, sorted EpCAM^+^ tumor cells by fluorescence-activated cell sorting (FACS) and measured *Ccl11* expression, which was higher in ATX-deficient PDAC cells (Fig. 5f). To test whether elevated *Ccl11* expression is sufficient to phenocopy ATX deficiency with respect to immune contexture, we generated *Ccl11*-overexpressing PDAC cells as well as empty vector controls (Extended Data Fig. 4c,d). *Ccl11* overexpression increased eosinophil recruitment into the PDAC microenvironment (Fig. 5g) and was sufficient to increase intratumor apoptosis and reduce tumor growth (Fig. 5h,i and Extended Data Fig. 4e). As a further test to mechanistically dissect the contribution of CCL11 signaling to our phenotype, we pharmacologically inhibited CCR3, the major receptor for CCL11. We inhibited CCR3 with GW766994, a selective antagonist of CCR3, administered by daily oral gavage to mice with established tumors and treated them for 7 d. Inhibition of CCR3 activity in ATX-depleted tumors led to significant rescue of tumor growth and reduced eosinophil infiltration compared to vehicle-treated, ATX-depleted tumors (Fig. 5j,k). We found no significant differences in *Ccr3* mRNA expression levels across sorted eosinophils from ATX-replete and ATX-depleted tumors (Fig. 5l) and note that GW766994 did not completely block eosinophil accumulation in the *Enpp2*-knockdown setting nor completely restore tumor growth.

These results prompted us to investigate the mechanism by which ATX–LPA signaling negatively regulates PDAC cell expression of *Ccl11*. As the AP-1 transcription factor c-Jun regulates *Ccl11* expression in other cellular contexts^44^, we measured c-Jun phosphorylation and nuclear trafficking and found both to be increased in ATX-deficient PDAC cells (Fig. 5m and Extended Data Fig. 4f–h), suggesting an unexpected negative regulation of c-Jun activity by ATX signaling in PDAC cells. We next measured phospho-c-Jun abundance in PDAC in vivo, which was increased in ATX-deficient tumors (Fig. 5n and Extended Data Fig. 4i), consistent with the negative regulation of PDAC cell-intrinsic AP-1 activity by ATX–LPA. To address the relevance of these findings to *Ccl11* expression, we performed chromatin immunoprecipitation for c-Jun and phospho-c-Jun in PDAC cells, which were more abundant at the *Ccl11* locus in the setting of ATX deficiency (Fig. 5o). As LPA canonically serves its functions through the activation of transmembrane LPA receptors (LPAR1–LPAR6)^13^, we assessed whether alterations in LPAR expression may underlie our observed phenotype. We found no significant differences in *Lpar1*–*Lpar6* transcript levels between control and ATX-deficient cells (Extended Data Fig. 4j). To understand the functional significance of LPA receptors in CCL11 expression, we pharmacologically inhibited LPA receptors before measuring *Ccl11* transcript expression and protein secretion. We treated PDAC cells with a combination of Ki6425 (LPAR1 and LPAR3 antagonist) and H2L5186303 (LPAR2 antagonist) to distinguish LPAR antagonism effects from ATX inhibition. Pharmacological inhibition of LPAR1–LPAR3 did not impact *Ccl11* gene expression levels or secretion levels, implicating the possible roles of LPAR4–LPAR6 in signaling downstream of ATX and LPA to suppress *Ccl11* expression (Extended Data Fig. 4k,l). Additionally, we observed no significant changes in mRNA expression levels of *Lpar1*–*Lpar6* across eosinophils isolated from ATX-replete and ATX-depleted tumors by FACS (Extended Data Fig. 4m). Together, these results indicate that ATX negatively regulates AP-1-mediated expression of CCL11 in PDAC cells, suppressing the recruitment of eosinophils into the tumor microenvironment (Fig. 5p).

## Discussion

PDAC is characterized, in part, by its dense, desmoplastic stroma, which is implicated in disease progression and resistance to therapy^45^. Inducible mouse models have demonstrated that among the earliest responses to oncogenic KRAS activation or extinction in the pancreatic epithelium is the expansion or contraction, respectively, of this stromal reaction^46–48^, linking the core oncogenic circuitry driving pancreatic tumorigenesis to desmoplasia. In light of evidence functionally linking PDAC cell-intrinsic mechanisms (including the KRAS effector MYC^49^, fatty acid sensor PPARδ^50,51^ and neutrophil chemoattractant CXCL1 (ref. ^11^)) to evolution of an immune-suppressive stroma, identifying putatively druggable cancer cell-intrinsic mechanisms that promote this immune-suppressive, treatment-resistant microenvironment has the potential to highlight previously uncharacterized targets for therapeutic intervention and bring much-needed improvement in outcomes to individuals with PDAC. Our prior work characterized a lipid metabolic switch in the PDAC stroma wherein pancreatic stellate cells, following differentiation to cancer-associated fibroblasts, secrete high levels of lysophosphatidylcholines; these lipids are hydrolyzed by cancer cell-derived ATX, resulting in mitogenic LPA signaling through LPA receptors and downstream activation of effector pathways implicated in proliferation and survival^24^. Beyond the significance of this tumor–stroma cross-talk for lipid metabolism and PDAC cell phenotypes, the present study demonstrates that PDAC cell-derived ATX promotes both cancer cell-intrinsic mechanisms relevant to the viability of RAS-transformed cells as well as microenvironmental regulation permissive of tumor growth.

The mechanisms linking ATX–LPA signaling to proliferation and cancer cell viability seem to be somewhat conserved across tissues and tumor types; for example, this axis regulates AKT activation in PDAC cells, as demonstrated here and previously^24^, with similar results in lung cancer^52^ and breast cancer^53^. However, regulation of the tumor immune microenvironment by cancer cell-derived ATX has only been demonstrated recently and exhibits clear tissue specificity with respect to the immune cell subsets impacted by this lipid signaling axis. Functional interactions between ATX–LPA signaling and T cell migration are well established^54^ and seem to be co-opted or perturbed in cancer. Although we show here that PDAC cell-derived ATX suppresses the infiltration of eosinophils into tumors, other groups have recently demonstrated clearly distinct responses to perturbation of ATX–LPA signaling in other tumor types. Specifically, this axis suppresses type I interferon signaling and abundance of antigen-experienced CD4^+^ and CD8^+^ T cells in the context of ovarian cancer^14^ and suppresses infiltration of CD8^+^ T cells in melanoma^18^, with potential impacts on immune contexture of other tumor types yet to be explored. Although T cell abundance was unaffected by ATX inhibition in our models, we note that this may reflect a limitation of the implantable, orthotopic PDAC models used here and that genetically engineered tumor models with slower growth kinetics may reveal additional links between ATX and immune cell abundance in PDAC. Although T cell abundance was unaffected by ATX inhibition in our assays, decreased B cell infiltration in pancreatic tumors was observed following inhibition of this signaling axis. This interaction warrants further investigation in light of compelling work implicating B cells in pancreatic tumor progression^55–58^. This work also calls into question the molecules governing eosinophil migration to the tumor microenvironment in the context of intact ATX signaling and in other tumor types. Although eotaxins regulate eosinophil migration under inflammatory conditions, the contributions of eotaxins to eosinophil migration in cancer have been largely unexplored, and expression of this chemokine family as well as other eosinophil migration mechanisms likely exhibits heterogeneity across the population.

The context dependency of the impact of ATX on the immune microenvironment raises the question of the relative significance of tissue context versus cancer cell state that dictates these differences. For example, whether the impact of ATX on the immune contexture of lung cancer would differ from its impact on pancreatic cancer lung metastases remains in question and may hold relevance for the potential development of combination therapies targeting ATX to foster efficacy of immunotherapies. This possibility gains relevance in light of a recent study demonstrating eosinophil-dependent activation of CD8^+^ T cells as a mechanism underlying response to immune checkpoint blockade in breast cancer^59^. In addition, as eosinophils have not been functionally investigated in PDAC previously, their potential significance for combination therapy as well as the mechanisms underlying their metastasis-restraining potential remain unclear and should be investigated in future studies. These findings motivate future efforts to uncover the downstream functions of eosinophils in PDAC and their potential implications for therapy.

## Methods

All experiments with mice and human-derived material were performed in accordance with local animal protection guidelines of Oregon Health & Science University or with approval of the Oregon Health & Science University Institutional Review Board, respectively. Further information on research design is available in the Nature Portfolio Reporting Summary linked to this article.

### Animals

All experiments were reviewed and overseen by the Institutional Animal Use and Care Committee at Oregon Health and Science University (OHSU), in accordance with National Institutes of Health guidelines for the humane treatment of animals. Male and female C57BL/6J (000664) mice from Jackson Laboratory were used for orthotopic transplant experiments. Congenitally eosinophil-deficient PHIL mice were a kind gift from A. Fryer (OHSU). *Rosa26-M2rtTA* mice were purchased from the Jackson Laboratory (006965). Male and female mice aged 8–12 weeks were used for all experiments. Littermate controls were used whenever possible. Animals included in tumor experiments were extensively assessed daily based on score sheets with criteria including body condition scoring and physical examination to prevent animal burden. Orthotopic tumors were grown to a maximum diameter of 1.0 cm based on institutional guidelines. Maximal burden was not exceeded with any animal.

### Human tissue samples

Human PDAC tissue arrays generated from human samples donated to the Oregon Pancreas Tissue Registry program with informed written consent (Institutional Review Board approval number IRB00003609), in accordance with full ethical approval by the OHSU Institutional Review Board, were kindly shared by R. Sears (OHSU). The tumor microarrays were either from OHSU (*n* = 47 primary PDAC cores, one single core per case) or were obtained commercially (US Biomax PA805c, *n* = 80, including 58 PDAC specimens, remaining from benign adjacent tissue, normal pancreas or other carcinomas of the pancreas; TissueArray.com PA811, pancreas adenocarcinoma tissue microarray with matched cancer-adjacent tissue, containing 25 cases of pancreas adenocarcinoma, remaining from cancer-adjacent tissue or other carcinomas of the pancreas; only instances of PDAC were scored here). Sections were scored, and eosinophil scores were determined by a board-certified pathologist (T.K.M.) for the maximum number of stained eosinophils per high-power field (×40 objective). For human samples, both males and females and those of any gender identity were included in data collection.

### Cell lines

The human pancreatic cancer cell line PaTu 8988T (RRID: CVCL_1847) was grown in DMEM containing 10% fetal bovine serum (FBS).

### Generation of stable cell lines

FC1245 PDAC cells were generated from a primary tumor in a *Kras*^LSL-G12D/+^; *Trp53*^LSL-R172H/+^; *Pdx1-cre* mouse and were kindly provided by D. Tuveson (Cold Spring Harbor Laboratory). The pSpCas9(BB)-2A-Puro(PX459) v2.0 plasmid (Addgene, 62988) was used to clone guide sequences targeting *Enpp2*, per the supplier’s protocol (sgRNA: TGGCCAGCGTATACAGATTA). The FC1245 PDAC cell line was transfected with PX459 control plasmid or plasmid containing the sgEnpp2 sequences and subjected to selection with 2 μg ml^–1^ puromycin for 4 d. Single-cell clones were expanded and screened for ATX expression by western blotting. The vectors containing shRNA targeting mouse *Enpp2* were purchased from Sigma. In brief, 15 μg of vector, together with 7.5 µg of each packaging vector (pMD2.G and psPAX2), were cotransfected into 293T cells. Supernatant-containing lentivirus particles were collected 48 and 72 h after transfection, passed through a 0.45-µm membrane filter and directly used to infect FC1245 mouse PDAC cells in the presence of 6 µg ml^–1^ polybrene. After 48 h, the infected cells were maintained in the medium with 2 µg ml^–1^ puromycin for 2 weeks before knockdown assessment.

All cell lines were routinely passaged in DMEM (Thermo Fisher Scientific) containing 10% FBS (HyClone), 25 mmol liter^–1^ glucose and 2 mmol liter^–1^ glutamine for no more than 25 to 30 passages.

### Pharmacological compounds

Dox was purchased from Millipore Sigma (24390-14-5), PF-8380 (12018) and GLPG1690 (25498) were purchased from Cayman Chemicals, and HA130 was purchased from Echelon Bioscience (B-0701). IL-4 (404-ML-010) and TNFα (410-MT-025) were purchased from R&D Systems. All pharmacological compounds were suspended and stored according to the manufacturer’s guidelines.

### Cell viability assays

PDAC cells were seeded in white-walled 96-well plates at 5 × 10^3^ cells per well in DMEM containing 10% FBS. The next day, cells were washed with PBS and treated as indicated with 10 μM HA130 (Echelon Bioscience, B-0701) or 4 μg ml^–1^ Dox (Sigma-Aldrich, D9891) at the time of cell seeding in serum-free DMEM. After 72 h or at the time points indicated in the manuscript, cells were lysed with CellTiter-Glo Luminescent Cell Viability Assay reagent (Promega), as per the manufacturer’s protocol. Luminescence was read using a GloMax plate reader.

### Gene expression analysis by RT–qPCR

DMEM containing 10% FBS was used to seed 5 × 10^5^ PDAC cells per well in six-well plates. Cells were treated with Ki16425 (Cayman, 10012659), H2L5186303 (Cayman, 14663) or HA130 (Echelon Bioscience, B-0701) for 24 h the following day, when pharmacological inhibitions were applicable. According to the manufacturer’s instructions, total RNA was isolated using Trizol (Thermo Fisher Scientific, 15596018). Using the iScript Reverse Transcription Supermix kit (Bio-Rad, 1708841), 1 g of RNA was reverse transcribed to produce cDNA. SYBR Green Supermix (Bio-Rad, 4367695) was used for RT–qPCR. Primer-Blast and the NCBI Nucleotide database were used to design gene-specific primer pairs. The 36B4 housekeeping gene was used to normalize relative gene expression. Following flow sorting, EpCAM^+^ mouse tumor cells from mouse PDAC tumors were collected in DMEM supplemented with 10% FBS and processed for subsequent RT–qPCR analysis with an Arcturus PicoPure RNA extraction kit (KIT0204). Primer sequences are provided in Supplementary Table 1.

### Orthotopic transplant/allograft model

The orthotopic transplant model used here was previously described^50^. D. Tuveson (Cold Spring Harbor Laboratory) kindly provided FC1245 PDAC cells generated from a *Kras*^LSL-G12D/+^; *Trp53*^LSL-R172H/+^; *Pdx1*-*cre* mouse primary tumor. We orthotopically transplanted 1,000 WT FC1245 mouse PDAC cells or 1,000 stably transduced FC1245 cells in 50% Matrigel into 8- to 10-week-old WT male or female C57BL/6J mice. Following institutional guidelines, mice were killed at humane end points (tumor area and tumor weight were measured), and pancreata were collected, sliced and flash-frozen in liquid nitrogen or immediately fixed in formalin.

### Dox induction of ENPP2 depletion in vivo

Following orthotopic transplantation of stably transduced FC1245 mouse PDAC cells into *Rosa26-M2-rtTA* mice, tumor establishment was verified by using ultrasound to measure tumor areas. For *Enpp2* gene depletion during PDAC tumor development in adult mice, tumor-bearing mice were treated with Dox for 7 d, after which, mice were killed, tumor areas and weights were recorded, and tumors were collected according to institutional guidelines. To determine dox-independent gene deletion, that is, promoter leakiness, mice were not treated. Dox was reconstituted in cell culture-grade DMSO (Sigma-Aldrich) at 100 mg ml^–1^, aliquoted and stored at −20 °C. To administer dox, dox was diluted to 2 mg ml^–1^ in autoclaved 10% sucrose-supplemented drinking water and was provided to mice in red water bottles, which was changed every other day.

### Siglec-F depletion

At day 12 following orthotopic transplantation with WT FC1245 mouse PDAC cells, male C57BL/6 mice were imaged by ultrasound to record tumor areas and were randomized into treatment groups, including mice that were treated with isotype-matched control IgG or Siglec-F-depleting antibody (20 μg of IgG or antibody per mouse; Siglec-F antibody, R&D Systems, MAB17061; control rat IgG antibody, R&D Systems, MAB006). Tumors were measured, and mice were killed after 10 d of treatment (Siglec-F or IgG intraperitoneal injections every alternate day, five doses total), and pancreata were collected, sliced and flash-frozen in liquid nitrogen or immediately fixed in formalin.

### Daily oral gavage with the CCR3 antagonist GW766994

Male C57BL/6J mice were treated daily for 7 consecutive days with 8 mg per kg (body weight) of the CCR3 selective antagonist GW766994 (MCE, HY-107051) by oral gavage, following institutional guidelines. Vehicle carrier was prepared according to the manufacturer’s guidelines (10% DMSO/90% corn oil).

### ENPP2 pharmacological inhibitor treatment

In this study, male C57BL/6J mice were anesthetized with ketamine and xylazine and injected with FC1199 cells (provided by D. Tuveson) or 4662 cells (provided by R. Vonderheide), both derived from primary PDAC cells in pure KPC C57BL/6J mice. Using the Vevo 770 imaging system, pancreata were imaged by high-resolution ultrasound 14 d after transplantation; mice were enrolled in the study when the tumor diameters reached 3–6 mm, and pretreatment tumor sizes were noted. The mice were given 30 mg per kg (body weight) ONO-8430506 dissolved in 7.5 mmol liter^–1^ NaOH twice daily or a vehicle control twice daily. Ten days after treatment, a Vevo 770 was used to measure post-treatment tumors. The mice were killed, and tissues were collected for analysis following institutional guidelines.

### Spectral flow cytometry

Orthotopic pancreas tumors were removed surgically and were checked to ensure that they were free of any contaminating tissue. Tumors were placed in 1.8 ml of base RPMI-1640 medium without FBS in a 24-well plate. Plates were kept cold on ice to preserve cell viability. Using surgical scissors, tumors were cut into pieces less than 1 mm^3^ in size within each well and were treated with 200 μl of digestion cocktail (10× solution: 0.25% collagenase I (STEMCELL Technologies, 07902), 1 mg ml^–1^ collagenase IV (STEMCELL Technologies, 07909), 10× collagenase/hyaluronidase (STEMCELL Technologies, 07912), 5 U ml^–1^ dispase (STEMCELL Technologies, 07913), 1 mg ml^–1^ DNase I (STEMCELL Technologies, 07900) and 1× Accumax Cell Dissociation Solution (Innovative Cell Technologies, AM105)) diluted to 1× in base RPMI-1640 medium (without FBS). Tumor samples were digested for 1 h at 37 °C while lightly shaking at 60–100 r.p.m. using an orbital plate shaker. After 1 h, the digestion reaction was neutralized by adding 1 ml of RPMI-1640 medium supplemented with 5% FBS and 2 mM EDTA to each well. The tumor digestion mix was pipetted onto a 40-µm cell strainer seated on top of a 50-ml centrifuge tube, and a 10-ml syringe plunger was used to mechanically disaggregate the tumor pieces through the strainer. The strainer was periodically flushed with 2 ml of supplemented RPMI-1640 medium (containing 5% FBS), which was repeated until the cell strainer was clear of tumor. Cell suspensions were centrifuged (5 min, 805*g*, 4 °C), supernatants were discarded, and pellets were resuspended in 2 ml of supplemented RPMI-1640 medium. Cells were then subjected to red blood cell lysis in ACK lysis buffer (Thermo Fisher Scientific) for 3 min at room temperature, washed in FACS buffer (PBS containing 2% FBS), pelleted and resuspended in FACS buffer for staining. Following red blood cell lysis, cells were incubated with anti-CD16/CD32 (BD Biosciences, 553141) for 2 min at room temperature to block Fc receptors and were then stained with antibodies for 30 min on ice. The antibodies used for flow cytometry experiments are listed in the Reporting Summary. A Cytek Aurora spectral analyzer was used to analyze tumor samples.

### Gating strategy for the full Cytek Aurora staining panel

CD45^+^ leukocytes were gated from live single cells (Zombie NIR was used as a live/dead dye). CD64^+^CD11b^+^ macrophages comprised a Ly6C^+^ inflammatory subset and two Ly6C^lo^ subsets, MHCII^hi^ or CD206^hi^. From the CD64^lo^ population, neutrophils were identified as CD11b^+^Ly6G^+^. In the Ly6G^−^ population, dendritic cells were defined as MHCII^+^CD11c^+^. Eosinophils were gated as Siglec-F^+^SSC^hi^ cells from the CD11c^lo^ subset, as were CD19^+^ B cells. Within the Ly6G^−^CD11c^lo^Siglec-F^−^CD19^−^ population, CD3^+^ T cells and subsets of CD4^+^ T cells and CD8^+^ T cells were identified. The CD4^+^ subset further contained a population of CD25^+^ regulatory T cells. CD3^−^ subsets were gated as NK1.1^+^ natural killer cells or CD11b^+^Ly6C^+^ inflammatory monocytes.

### FACS gating strategy for isolation of pure eosinophil populations from tumors

Isolated cells were incubated on ice with anti-mouse CD16/CD32 (clone 2.4G2, BD Biosciences) to block Fc receptors and then incubated on ice for 30 min with a mix of fluorochrome-conjugated anti-mouse antibodies. Flow cytometry data were analyzed using FlowJo. The gating strategy was based on side and forward scatter, followed by singlets discrimination and then gating on all lymphocytes defined as CD45^+^ and all epithelial cells defined as EpCAM^+^. We then gated on all CD45^+^ events to define all myeloid cells as CD11b^+^. CD11b^+^ myeloid cells were further analyzed to gate eosinophils and neutrophils based on Siglec-F (eosinophils) and Ly6G (neutrophils) expression.

### Enzyme-linked immunosorbent assay (ELISA)

In total, 3.5 × 10^6^ FC1245 PDAC cells were seeded in 10-cm plates with DMEM (Thermo Scientific, 11965126) containing 10% VWR Seradigm FBS (VWR, 97068-085) and 1 mM sodium pyruvate (Thermo Scientific, 11360070). For pharmacological treatments, cells were washed with PBS and treated for 24 h in serum-free DMEM with 10 μM Ki16425 (Cayman, 10012659), H2L5186303 (Cayman, 14663) or HA130 (Echelon Bioscience, B-0701). The drugs were reconstituted in DMSO. A sandwich immunoluminometric assay was used to measure the level of mouse CCL11 secreted 24 h after conditioned medium was added (mouse CCL11 Quantikine ELISA kit; R&D Systems, MME00). A Cytiva 28932358 Ultracel 3-kDA filter unit was used to concentrate the conditioned medium samples before ELISA.

### TUNEL assay

TUNEL assays were performed using the Click-iT TUNEL Alexa Fluor 488 imaging assay according to the manufacturer’s instructions (C10245).

### Western blotting

PDAC cells were serum starved for 48 h and treated as described in the text. In accordance with the manufacturer’s instructions, subcellular fractions were prepared using Thermo Fisher Scientific’s NE-PER Nuclear and Cytoplasmic Extraction reagents. Bicinchoninic protein assay kits (Pierce) were used to measure protein concentrations. We loaded equal amounts of protein in each lane, separated the proteins on a 4–12% Bis-Tris NuPAGE gel (Invitrogen) and transferred the proteins to PVDF membranes. The following primary antibodies and infrared secondary antibodies were used to probe the membranes: IRDye 700 goat anti-rabbit IgG and IRDye 800 goat anti-mouse IgG (LI-COR Biosciences). LI-COR Biosciences’ Image Studio software (LI-COR) was used to quantify protein bands using infrared signals detected by the Odyssey CLx infrared imaging system.

### Two-plex fluorescence in situ hybridization

Thermo Fisher Scientific provided a ViewRNA ISH Tissue Assay kit (two plex) for use on mouse PDAC tumor tissue samples. Briefly, samples were first permeabilized with controlled protease digestion, followed by incubation with proprietary probe-containing solution, according to the manufacturer’s instructions. During incubation, samples had to remain fully submerged. After hybridization with the probe, samples were washed, followed by sequential hybridization with the preamplifier and amplifier DNA. In accordance with the manufacturer’s instructions, hybridizations were performed with the preamplifier, amplifier and fluorophore. Mounting medium (DAKO ultramount) was used to mount samples.

### Immunohistochemistry

#### Cells

Cells were fixed in 4% paraformaldehyde for 15 min at room temperature, washed three times in PBS and permeabilized in 0.1% Triton X-100 for 10 min. Coverslips were blocked in blocking solution for 1 h at room temperature before transferring them to a carrier solution containing diluted antibodies (see Reporting Summary). The coverslips were incubated for 3 h at room temperature and then washed five times with PBS for 5 min each. Following the primary antibody incubation, secondary Alexa Fluor-conjugated antibodies were added to the coverslips for 1 h at room temperature diluted in the same carrier solution (1:400). Incubation of the secondary antibody was followed by five 5-min washes in PBS and mounting with Vectashield DAPI mounting medium.

#### Mouse and human tissue samples

A protocol for anesthesia and euthanasia was followed according to institutional guidelines. After surgical removal of pancreatic tumors, the samples were drop-fixed overnight in 4% paraformaldehyde for up to 48 h. The OHSU Histopathology Core performed paraffin embedding, sectioning and hematoxylin and eosin staining on tissue samples. We deparaffinized mouse and human tissue sections in an ethanol gradient and rehydrated them in PBS. A block solution (Aqua Block buffer from Abcam) was applied to tissue samples for 2 h at room temperature following antigen retrieval, and they were then transferred to a carrier solution containing diluted antibodies (see Reporting Summary). After overnight incubation at 4 °C, sections were washed five times in PBS for 5 min each, and secondary Alexa Fluor-conjugated antibodies were added for 1 h at room temperature in the same carrier solution (1:400). A Vectashield mounting medium containing DAPI was used to mount the sections after washing them five times in PBS for 5 min each.

#### Eosinophil staining for mouse and human tissue sections

Tissue eosinophils were detected using an immunohistochemical stain against mouse eosinophilic MBP. Endogenous peroxidase activity was quenched using a 0.3% hydrogen peroxide in methanol solution. The tissue was treated with pepsin for 10 min at 37 °C (DIGEST-ALLTM 3, Zymed Laboratories). Nonspecific binding was blocked using 3% horse serum in PBS for 2 h at room temperature, followed by the addition of rabbit anti-mouse MBP (~1:1,000; kindly provided by E. Jacobsen, Mayo Clinic Scottsdale), which was allowed to incubate overnight at 4 °C. Slides were incubated with alkaline phosphatase-conjugated anti-rabbit (1:250) for 40 min at room temperature. Development of the alkaline phosphatase reaction was achieved by incubating slides with Fast Red substrate (Vector Laboratories) for 5 min at room temperature and then counterstaining with nuclear hematoxylin. Quantification of positive cells was performed using Leica Aperioscope imaging software.

#### Wright’s Giemsa differential stain

Cell smears were quick fixed in methyl alcohol for 1 min. Fixed smears were submerged in Wright–Giemsa stain (Abcam, ab245888) for 3 min, followed by submersion in phosphate buffer at pH 6.8 for 1 min. Stained slides were rinsed in deionized water for 1 min (five total washes), air dried, submerged in Citrisolv and cover slipped using a permanent mounting medium. Eosinophils stained bright red.

### Microscopy

#### Confocal microscopy

Imaging of fluorescence staining was performed by confocal imaging of fixed cells and tissues with a laser-scanning confocal inverted microscope (Carl Zeiss, LSM 880), and a ×40/1.1-NA water objective or ×63/1.4-NA oil objective was used to image the samples. A Zeiss AxioScan automated slide-scanning microscope was used to acquire ×20 tiled images of entire hematoxylin and eosin-stained mouse tumor sections.

#### Slide scanning

Whole-slide scanning was performed at the OHSU imaging core using a Zeiss Axioscan 7 Slide Scanner.

#### Image analysis

A global thresholding method was used to analyze immunofluorescence images using ImageJ software. In an image, we were able to separate fluorescently labeled pixels from the background by applying global thresholding. The global thresholding technique selects a value cutoff at which pixels below that value were considered one class, and pixels above that value were considered another class. Using this technique, we were able to distinguish between two classes of pixels in an image, referred to as ‘foreground’ pixels and ‘background pixels’. The pixels classified as the foreground, or segmented pixels, in our application were also called fluorescent pixels because they are considered to carry the fluorescent signal of the image. We used ImageJ’s default image segmentation algorithm, which is the original method of autothresholding. Binaries 8-bit and 16-bit images using Default (histogram-derived algorithm). The positive pixel count per field of view was analyzed using Aperio Imagescope software for whole-slide scanned images.

#### TEM

Cell pellets were fixed in glutaraldehyde and formaldehyde in 0.1 M sodium cacodylate buffer and coated in 4% low-melting-point agarose. A Pelco Biowave Pro+ microwave was used to rinse samples in sodium cacodylate for 40 s at 150 W three times, and the samples were then stained with 2% osmium tetroxide and 1.5% potassium ferricyanide for 3 min at 100 W and 2 min at 0 W. Aqueous uranyl acetate (1%) was used to stain samples at 0, 100 and 150 W for 2 min each. After three rinses in water, samples were dehydrated in an acetone series (50, 75, 95 and 100 twice) for 40 s at 150 W and infiltrated with a 1:1 resin:acetone mix, followed by pure resin infiltrations four times for 3 min. Samples were polymerized in an oven at 60 °C for 36 h.

Mouse tumors were fixed in 0.1 M sodium cacodylate buffer with 2.5% glutaraldehyde and 2.5% formaldehyde. Fixed tumors were chopped into pieces 1 mm^3^ in size and placed in mPrep/s capsules, which were connected to an ASP-2000. After three rinses in sodium cacodylate buffer, 2% osmium tetroxide and 1.5% potassium ferricyanide were applied, followed by three rinses in water, application of 1% aqueous uranyl acetate and three water rinses. The specimens were then dehydrated in acetone (50, 75, 85, 95 and 100% three times) and infiltrated with resin and acetone, followed by three rinses in pure resin. Staining was performed at 15 viscosity with 2-s aspiration holds, which was repeated 200 times. We performed 100 cycles of rinse and dehydration with a viscosity of 15. Increasing times were used during infiltration; 1:1 resin:acetone with a viscosity of 15 and 100 cycles, 3:1 resin:acetone with a viscosity of 15, aspiration hold of 10 s and 100 cycles. Infiltration was performed with a viscosity of 30 for the first pure resin step and 150 or 999 for the second pure resin step. Samples were polymerized for 36 h at 60 °C after capsules were removed. An FEI Tecnai T12 transmission electron microscope equipped with an AMT NanoSprint12S-B camera at 120 kV was used to image samples after polymerization.

### Chromatin immunoprecipitation

Chromatin immunoprecipitation and sequencing were performed as described previously^51^. PDAC cells were fixed in 1% formaldehyde; the nuclei were extracted and lysed in 1% SDS, 10 mM EDTA, 50 mM Tris-HCl (pH 8.0) and protease inhibitors; and the chromatin fragments were sheared with a Diagenode Bioruptor to 200 to 1,000 base pairs in size. An antibody to c-Jun (Cell Signaling Technology, 9165) was used to immunoprecipitate chromatin. Chromatin immunoprecipitation–quantitative PCR confirmed the gain of c-Jun binding at the *Ccl11* gene promoter sequences. A control intergenic region of the genome was used to normalize enrichment values. Primer sequences are described in Supplementary Table 1.

### Antibodies

Antibody details are available in the Nature Portfolio Reporting Summary linked to this article.

### Plasmids

Lentiviral mouse *Enpp2* shRNA constructs were purchased from Sigma-Aldrich Mission shRNA glycerol stocks (TRCN0000080829, TRCN0000080830, TRCN0000080831 and TRCN0000080832). Eotaxin-1 lentiviral overexpression plasmid was purchased from Origene (MR200278L4). ENPP2 rescue plasmid was purchased from Origene (MR223207L4, mouse-tagged ORF clone). *CCL11* shRNA plasmids were purchased from Origene (TR519095, four unique 29-mer shRNA constructs).

### Primer sequences

Gene expression analyses were conducted with the primer sequences provided in Supplementary Table 1.

#### Statistics and reproducibility

No statistical methods were used to predetermine sample sizes. The experiments were not randomized. A minimum number of mice was selected based on preliminary studies, with an effort to achieve a minimum of *n* = 3, mostly *n* = 5–10, mice per treatment group. Age-matched mice were selected for experiments. Researchers performing experiments with the mouse models were not blind to the experimental hypothesis. For histological staining quantitation, analyses were performed in a blinded fashion. For batch-processed images, image analyses were performed in an unbiased way using image analysis software. Some western blots and RT–qPCR assays were performed by a researcher blind to the experimental hypothesis. Animals were excluded if an animal needed to be removed from an experiment early to prevent disease burden. All experiments were performed and reliably reproduced at least two independent times.

GraphPad Prism 9 was used to generate graphs and for statistical analyses. Groups were tested for normality. Statistical significance was calculated for two unmatched groups by unpaired, two-sided *t*-tests with Welch’s correction or Mann–Whitney *U*-test. One- or two-way ANOVAs were used for more than two groups as specified, followed by Tukey’s multiple comparisons tests. Datasets are presented as mean ± s.e.m. *P* values under 0.05 were considered significant. Data distribution was assumed to be normal, but this was not formally tested.

### Reporting summary

Further information on research design is available in the Nature Portfolio Reporting Summary linked to this article.

## Supplementary information

Reporting Summary

Supplementary Table 1 Table for primer sequences.

## Source data

Source Data Fig. 1 Statistical source data.

Source Data Fig. 1 Unprocessed western blots and/or gels.

Source Data Fig. 2 Source data.

Source Data Fig. 3 Source data.

Source Data Fig. 4 Source data.

Source Data Fig. 5 Statistical source data.

Source Data Fig. 5 Unprocessed western blots and/or gels.

Source Data Extended Data Fig. 1 Source data.

Source Data Extended Data Fig. 2 Source data.

Source Data Extended Data Fig. 3 Source data.

Source Data Extended Data Fig. 4 Statistical source data.

Source Data Extended Data Fig. 4 Unprocessed western blots and/or gels.

## Data Availability

Source data for Figs. 1–5 and Extended Data Figs. 1–4 have been provided as source data files. All other data supporting the findings of this study are available from the corresponding author on reasonable request.

## Code availability

No code was generated/used in the preparation of this paper.
