# Author Correction: Autotaxin–lysolipid signaling suppresses a CCL11–eosinophil axis to promote pancreatic cancer progression

**DOI:** 10.1038/s43018-025-01035-9

**Published:** 2025-07-23

**Authors:** Sohinee Bhattacharyya, Chet Oon, Luis Diaz, Holly Sandborg, Erin S. Stempinski, Michelle Saoi, Terry K. Morgan, Claudia S. López, Justin R. Cross, Mara H. Sherman

**Affiliations:** 1 Department of Cell, Developmental & Cancer Biology, Oregon Health & Science University, Portland, OR USA; 2 Cancer Biology & Genetics Program, Memorial Sloan Kettering Cancer Center, New York, NY USA; 3 Multiscale Microscopy Core Facility, Oregon Health & Science University, Portland, OR USA; 4 Donald B. and Catherine C. Marron Cancer Metabolism Center, Memorial Sloan Kettering Cancer Center, New York, NY USA; 5 Department of Pathology, Oregon Health & Science University, Portland, OR USA; 6 Department of Biomedical Engineering, Oregon Health & Science University, Portland, OR USA

**Keywords:** Cancer microenvironment, Pancreatic cancer, Tumour immunology, Cancer

Correction to: *Nature Cancer* 10.1038/s43018-023-00703-y, published online 9 January 2024.

In the version of the article initially published, the right panel in Fig. 2a was incorrect as it showed data for “–Dox” rather than “+Dox”. This has now been replaced with the correct flow cytometry dot plot, as seen in Fig. 1. Additionally, in the second row, first panel of Extended Data Fig. 2a the axis labels were incorrect (“SSC-A”, “CD45”) and have now been amended to “Ly6C” and “NK1.1”. These corrections have been made to the HTML and PDF versions of the article.

**Fig. 1:**
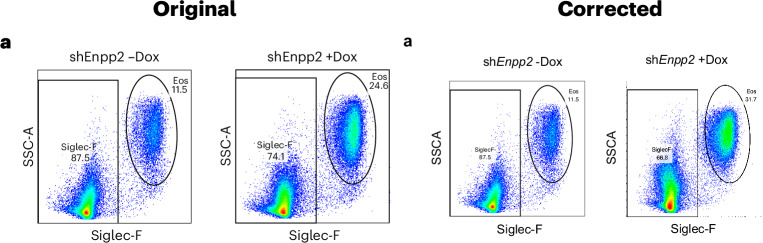
**Original and corrected Fig. 2a**.

