# Supplementary material for: Autotaxin–lysolipid signaling suppresses a CCL11–eosinophil axis to promote pancreatic cancer progression
Source: Nat Cancer. 2024 Jan 9;5(2):283–98. doi: 10.1038/s43018-023-00703-y (PMC10899115; doi:10.1038/s43018-023-00703-y)
Supplement: Supplementary file 1 — Reporting Summary [file 43018_2023_703_MOESM1_ESM.pdf]

nature portfolio

Corresponding author(s): Mara H. Sherman

Last updated by author(s): Nov 22, 2023

Reporting Summary

Nature Portfolio wishes to improve the reproducibility of the work that we publish. This form provides structure for consistency and transparency in reporting. For further information on Nature Portfolio policies, see our Editorial Policies and the Editorial Policy Checklist.

Statistics

For all statistical analyses, confirm that the following items are present in the figure legend, table legend, main text, or Methods section.

n/a	Confirmed	
☐	☒	The exact sample size (n) for each experimental group/condition, given as a discrete number and unit of measurement
☐	☒	A statement on whether measurements were taken from distinct samples or whether the same sample was measured repeatedly
☐	☒	The statistical test(s) used AND whether they are one- or two-sided<br>*Only common tests should be described solely by name; describe more complex techniques in the Methods section.*
☐	☒	A description of all covariates tested
☐	☒	A description of any assumptions or corrections, such as tests of normality and adjustment for multiple comparisons
☐	☒	A full description of the statistical parameters including central tendency (e.g. means) or other basic estimates (e.g. regression coefficient) AND variation (e.g. standard deviation) or associated estimates of uncertainty (e.g. confidence intervals)
☐	☒	For null hypothesis testing, the test statistic (e.g. F, t, r) with confidence intervals, effect sizes, degrees of freedom and P value noted<br>*Give P values as exact values whenever suitable.*
☒	☐	For Bayesian analysis, information on the choice of priors and Markov chain Monte Carlo settings
☒	☐	For hierarchical and complex designs, identification of the appropriate level for tests and full reporting of outcomes
☒	☐	Estimates of effect sizes (e.g. Cohen's d, Pearson's r), indicating how they were calculated

Our web collection on statistics for biologists contains articles on many of the points above.

Software and code

Policy information about availability of computer code

Data collection	BD Fortessa, Cytek Aurora, Leica Biosystems Aperio scanner, Leica Biosystems Arial scanner
Data analysis	Graphpad Prism v9, QuPath v0.2.3, FlowJo v9

For manuscripts utilizing custom algorithms or software that are central to the research but not yet described in published literature, software must be made available to editors and reviewers. We strongly encourage code deposition in a community repository (e.g. GitHub). See the Nature Portfolio guidelines for submitting code & software for further information.

Data

Policy information about availability of data

All manuscripts must include a data availability statement. This statement should provide the following information, where applicable:

- Accession codes, unique identifiers, or web links for publicly available datasets
- A description of any restrictions on data availability
- For clinical datasets or third party data, please ensure that the statement adheres to our policy

Source data for Fig. 1-5 and Extended Data Fig. 1-4 have been provided as Source Data files. All other data supporting the findings on this study are available from the corresponding author on reasonable request.

Research involving human participants, their data, or biological material

Policy information about studies with human participants or human data. See also policy information about sex, gender (identity/presentation), and sexual orientation and race, ethnicity and racism.

Reporting on sex and gender	Both males and females and those of any gender identity were included in specimen collection
Reporting on race, ethnicity, or other socially relevant groupings	Specimen collection was inclusive of participants of any race, ethnicity, or other socially relevant grouping. These personal details were not known to the authors of this study.
Population characteristics	Age of at least 18 and with a diagnosis of pancreatic ductal adenocarcinoma
Recruitment	All patients undergoing treatment for pancreatic ductal adenocarcinoma were given the option to participate and contribute to the Oregon Pancreas Tissue Registry, with a consent form provided by their physician for de-identified tissues to be used for retrospective analyses as performed in this study.
Ethics oversight	Oregon Health & Science University Institutional Review Board (approved protocol #3609)

Note that full information on the approval of the study protocol must also be provided in the manuscript.

Field-specific reporting

Please select the one below that is the best fit for your research. If you are not sure, read the appropriate sections before making your selection.

☒ Life sciences ☐ Behavioural & social sciences ☐ Ecological, evolutionary & environmental sciences

For a reference copy of the document with all sections, see nature.com/documents/nr-reporting-summary-flat.pdf

Life sciences study design

All studies must disclose on these points even when the disclosure is negative.

Sample size	No statistical methods were used to pre-determine sample size. Sample sizes were estimated based on preliminary experiments, with an effort to achieve a minimum of n=3, mostly n=5-10 mice per treatment group
Data exclusions	No data were excluded throughout the study.
Replication	All experiments were reliably reproduced and were performed at least 2 independent times.
Randomization	Mice with matched sex and age are randomized into different treatment groups or experimental arms.
Blinding	For histological analysis, samples were analysed in a blinded fashion when scored by hand, or were batch analysed in an unbiased manner using QuPath software.

Reporting for specific materials, systems and methods

We require information from authors about some types of materials, experimental systems and methods used in many studies. Here, indicate whether each material, system or method listed is relevant to your study. If you are not sure if a list item applies to your research, read the appropriate section before selecting a response.

Materials & experimental systems

n/a	Involved in the study	
☐	☒	Antibodies
☐	☒	Eukaryotic cell lines
☒	☐	Palaeontology and archaeology
☐	☒	Animals and other organisms
☒	☐	Clinical data
☒	☐	Dual use research of concern
☒	☐	Plants

Methods

n/a	Involved in the study	
☒	☐	ChIP-seq
☐	☒	Flow cytometry
☒	☐	MRI-based neuroimaging

Antibodies

Antibodies used	Antibodies used in the manuscript are as follows: Siglec-F antibody (R&D Systems MAB17061), control rat IgG antibody (R&D Systems

Antibodies used	MAB006), CD16/CD32 antibody (1:200; BD Biosciences, 553141), IRDye 700 goat anti-rabbit lgG or IRDye 800 goat anti-mouse lgG (1:1000; LI-COR Biosciences), MBP (1:500) and EPX (1:400) antibodies (kindly provided by Dr. Elizabeth Jacobsen), c-Jun (1:400; Cell Signaling Technology #9165), p-c-Jun (1:200; Cell Signaling Technology 3270), CD45 PerCPCY5.5 (1:200; BIOLEGEND 103131), CDllb BV570 (1:200; BIOLEGEND 101233), CD64 PE DAZZLE594 (1:200; BIOLEGEND 139319), LY6C AF700 (1:200; BIOLEGEND 128023), CD206 AF647 (1:200; BIOLEGEND 141711), CD301 APC (1:200; BIOLEGEND 145707), MHCII PACIFIC BLUE (1:200; BIOLEGEND 107619), LY6G BV711 (1:200; BIOLEGEND 127643), CDllc BV785 (1:200; BIOLEGEND 117335), Siglec F BV421 (1:200; BIOLEGEND 155509), CD19 PE (1:200; BIOLEGEND 115507), CD3E PECy5 (1:200; BIOLEGEND 100309), TCRB BV605 (1:200; BIOLEGEND 109241), NKl.1 BV650 (1:200; BIOLEGEND 108735), CD4 APCCY7 (1:200; BIOLEGEND 100525), CD8a BV510 (1:200; BIOLEGEND 100751), CD25 PE-CY7 (1:200; BIOLEGEND 102015), PDl PERCPEFLUOR710 (1:200; THERMO 46998582), Pan-KRT (Cll) mouse mAb (1:200; Alexa Fluor 647 Conjugate; Cell Signaling Technology, 4528), Cytokeratin Pan Type 1/11 Antibody Cocktail (1:500; Thermo Fisher Scientific, MA5-13156), Smooth muscle actin mouse mAb (1:500; 1A4 [asm-1] lnvitrogen, MA5-11547), pc-JUN rabbit mAb (1:200; Cell Signaling Technology, 3270), Ki-67 (1:400; D3B5, Cell Signaling Technology, 12202); Cleaved Caspase-3 rabbit mAb (1:400; Cell Signaling Technology, 9661); pAKT rabbit mAb (1:400; 736E11, Cell Signaling Technology, 3787), ENPP2 rabbit polyclonal antibody (1:200; Abcam, ab140915); phospho-Akt (1:200; Ser473; D9E) XP rabbit mAb (Cell Signaling Technology, 4060); Akt (pan; 40D4) rabbit mAb (1:400; Cell Signaling Technology, 2920); HSC 70 mouse antibody (1:500; Santa Cruz, SC7298); phoshpho cJUN rabbit mAb (1:200; Cell Signaling Technology, 3270); total cJUN rabbit mAb (1:400; Cell Signaling Technology, 9165).
Validation	All the antibodies are validated for the use of immunofluorescence, immunohistochemistry, chromatin immunoprecipitation, and histological analyses. See manufacturer's website for references.

Eukaryotic cell lines

Policy information about cell lines and Sex and Gender in Research

Cell line source(s)	Human pancreatic cancer cell line PA-TU-8988T was obtained from ATCC. FC1245 mouse PDAC cells were generated from a primary tumor in a KrasLSL-G12D/+;Trp53LSL-R172H/+;Pdx1-Cre mouse and kindly provided by Dr. David Tuveson (Cold Spring Harbor Laboratory). All FC1245 sgEnpp2 and shEnpp2 clones were generated by authors as described in Methods.
Authentication	Knockdown and knockout cell lines were validated by Western blot.
Mycoplasma contamination	Cell lines were tested for mycoplasma at least once monthly.
Commonly misidentified lines (See ICLAC register)	No commonly misidentified lines were used.

Animals and other research organisms

Policy information about studies involving animals; ARRIVE guidelines recommended for reporting animal research, and Sex and Gender in Research

Laboratory animals	Mouse strains C57BL/6J, Rosa26-rtTA-M2, and PHIL were used at 8-12 weeks of age (males and females)
Wild animals	Study did not involve wild animals.
Reporting on sex	Male and female mice were used.
Field-collected samples	Study did not involve samples collected from the field.
Ethics oversight	Oregon Health & Science University Internal Animal Care and Use Committee

Note that full information on the approval of the study protocol must also be provided in the manuscript.

Plants

Seed stocks	*Report on the source of all seed stocks or other plant material used. If applicable, state the seed stock centre and catalogue number. If plant specimens were collected from the field, describe the collection location, date and sampling procedures.*
Novel plant genotypes	*Describe the methods by which all novel plant genotypes were produced. This includes those generated by transgenic approaches, gene editing, chemical/radiation-based mutagenesis and hybridization. For transgenic lines, describe the transformation method, the number of independent lines analyzed and the generation upon which experiments were performed. For gene-edited lines, describe the editor used, the endogenous sequence targeted for editing, the targeting guide RNA sequence (if applicable) and how the editor was applied.*
Authentication	*Describe any authentication procedures for each seed stock used or novel genotype generated. Describe any experiments used to assess the effect of a mutation and, where applicable, how potential secondary effects (e.g. second site T-DNA insertions, mosiacism, off-target gene editing) were examined.*

Flow Cytometry

Plots

Confirm that:

☒ The axis labels state the marker and fluorochrome used (e.g. CD4-FITC).

☒ The axis scales are clearly visible. Include numbers along axes only for bottom left plot of group (a 'group' is an analysis of identical markers).

☒ All plots are contour plots with outliers or pseudocolor plots.

☒ A numerical value for number of cells or percentage (with statistics) is provided.

Methodology

Sample preparation	Samples were prepared from primary murine pancreatic tumors, with tissue dissociation, staining, and analysis performed on the same day. Dissociation and staining details are provided in Methods.
Instrument	Cytek Aurora or BD Fortessa
Software	FlowJo v9
Cell population abundance	Eosinophils were 15-30% of the Siglec-F+ SSChi cells from the CDllclo subset.
Gating strategy	CD45+ leukocytes were gated from live single cells (Zombie NIR was used as live/dead dye). CD64+ CDllb+ macrophages comprised a Ly6C+ in flammatory subset and two Ly6Clo subsets, MHCllhi or CD206hi. From the CD641o population, neutrophils were identified as CDllb+ Ly6G+. In the Ly6G− population, dendritic cells were defi ned as MHCII+ CDllc+. Eosinophils were gated as Siglec-F+ SSChi cells from the CDllclo subset, as were CD19+ B cells. Within the Ly6G− CDllclo Siglec-F− CD19− population, CD3+ T cells, and subsets of CD4+ T cells and CDS+ T cells were identifi ed. The CD4+ subset further contained a population of CD25+ Treg cells. CD3− subsets were gated as NKl.l+ NK cells or CDllb+ Ly6C+ inflammatory monocytes.

☒ Tick this box to confirm that a figure exemplifying the gating strategy is provided in the Supplementary Information.
