# Supplementary material for: Autotaxin–lysolipid signaling suppresses a CCL11–eosinophil axis to promote pancreatic cancer progression
Source: Nat Cancer. 2024 Jan 9;5(2):283–98. doi: 10.1038/s43018-023-00703-y (PMC10899115; doi:10.1038/s43018-023-00703-y)

Figure 1a. Western blots depicting ENPP2 abundance in FC1245 sgRNA mediated loss-of-function PDAC cells

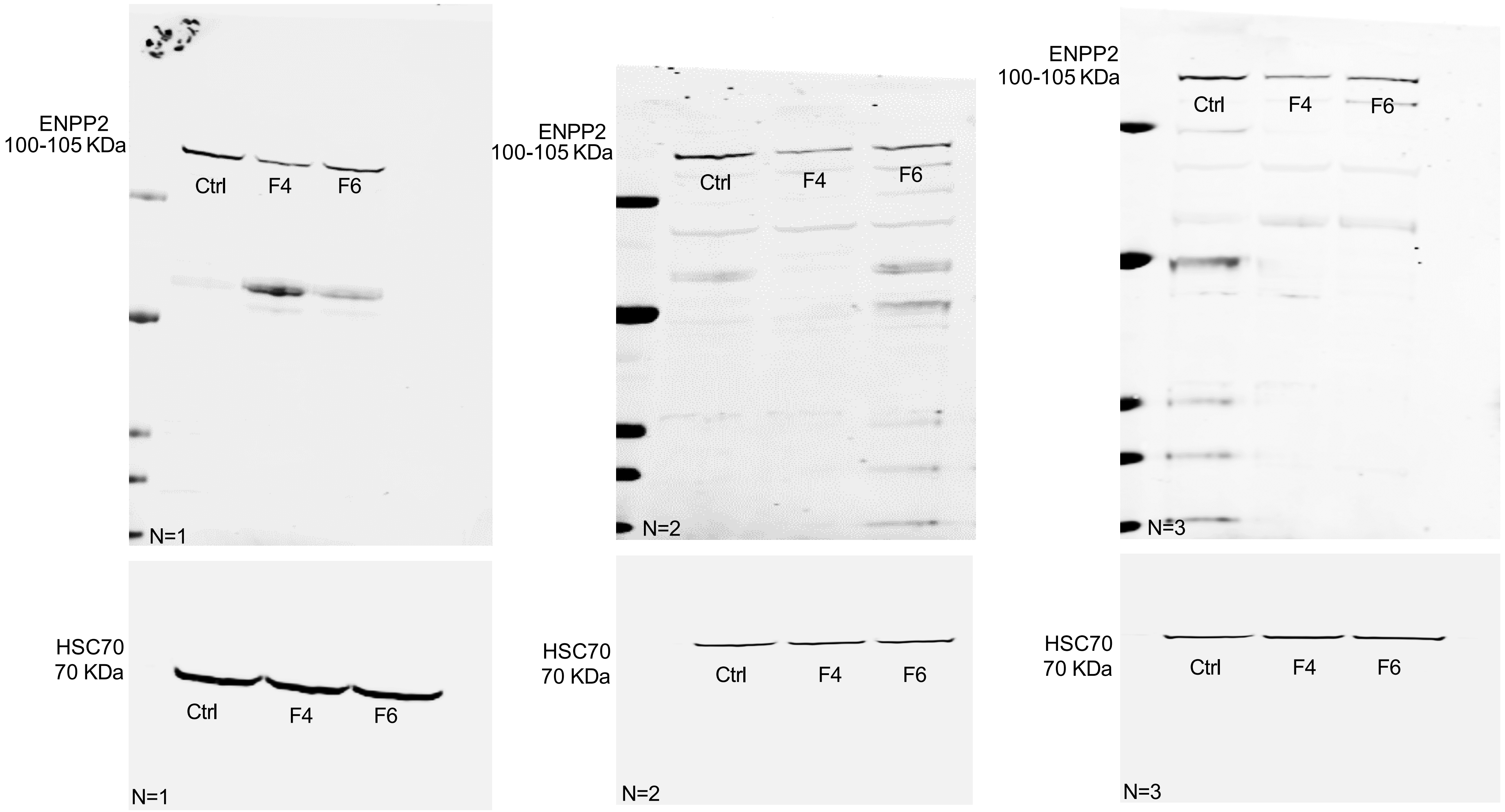

Figure 1a. Western blots depicting ENPP2 abundance in FC1245 Dox inducible shRNA mediated loss-of-function PDAC cells

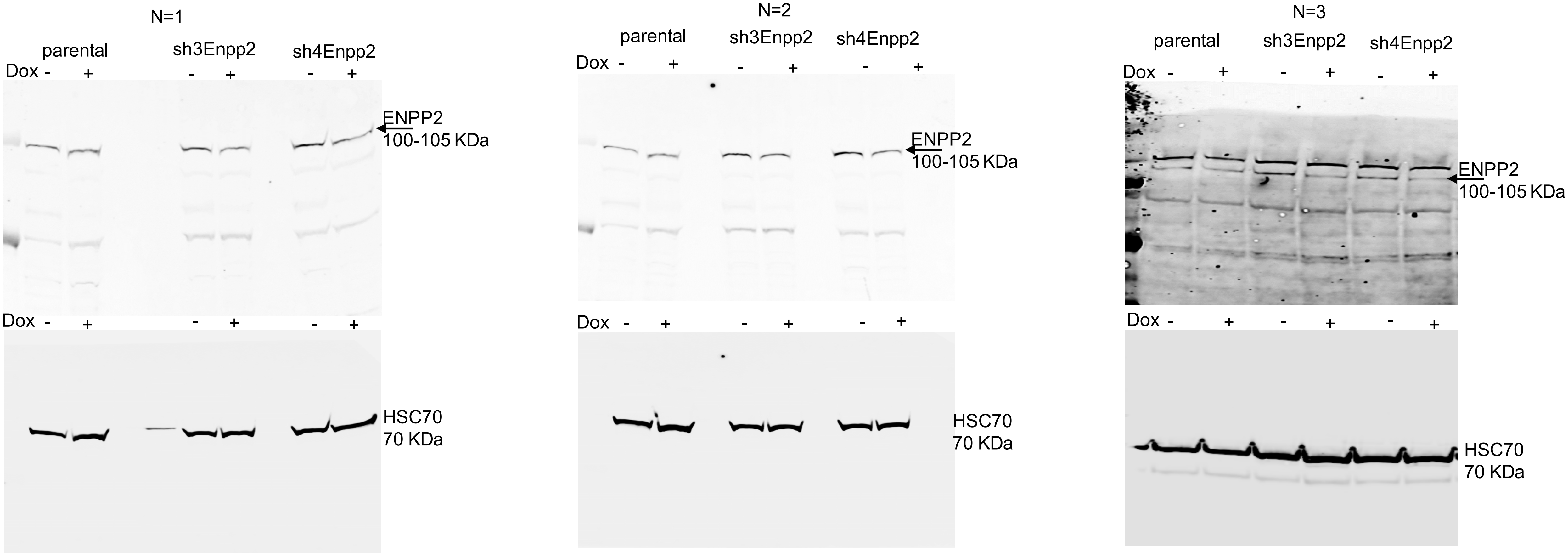

Figure 1i. Western blots depicting ENPP2(ATX) abundance in FC1245 sgCtrl, sgEnpp2, and reconstituted Enpp2 cell lysates

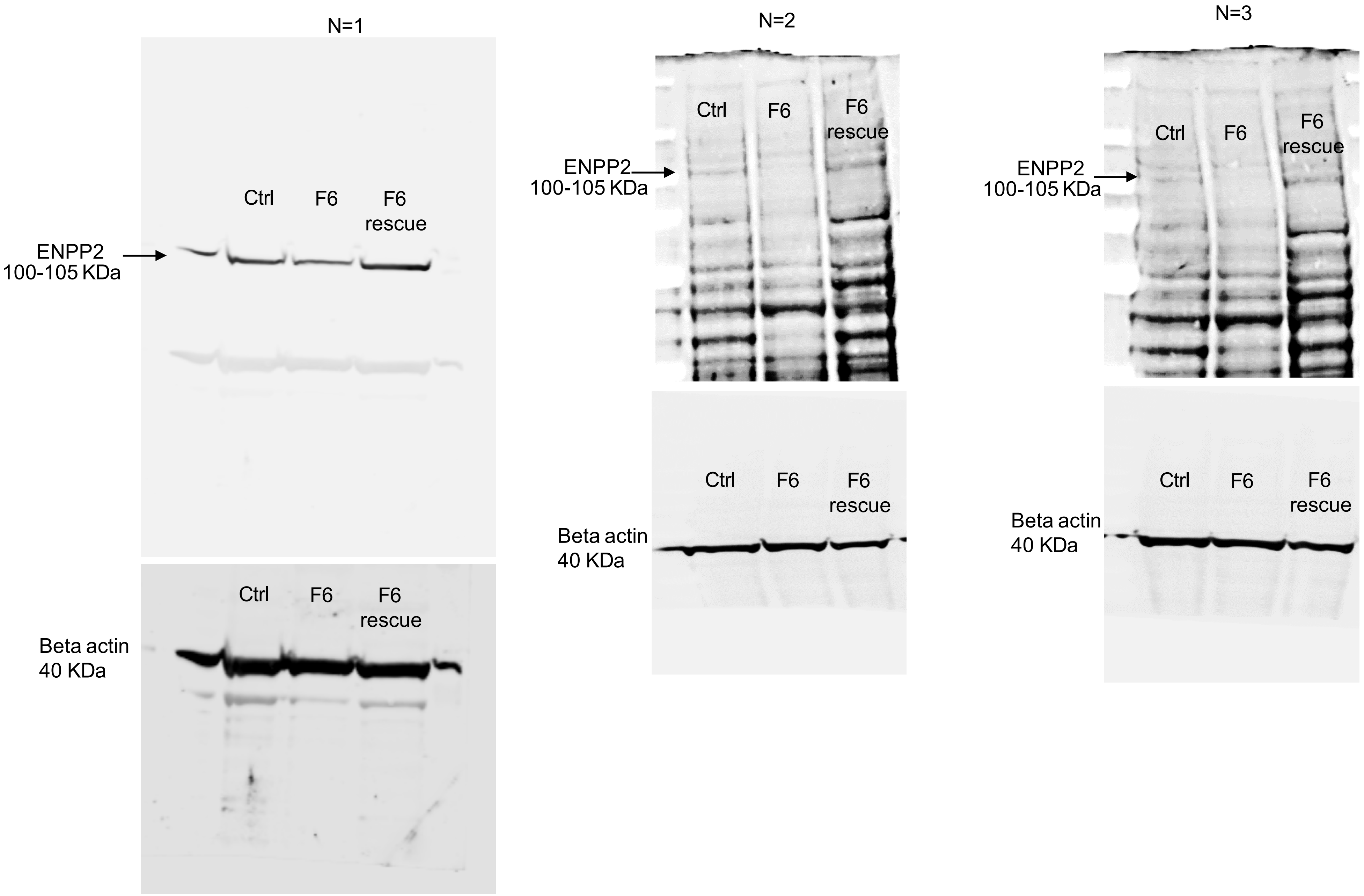

Supplement: Supplementary file 4 — Unprocessed western blots and/or gels. [file 43018_2023_703_MOESM4_ESM.pdf]
