# Supplementary material for: Autotaxin–lysolipid signaling suppresses a CCL11–eosinophil axis to promote pancreatic cancer progression
Source: Nat Cancer. 2024 Jan 9;5(2):283–98. doi: 10.1038/s43018-023-00703-y (PMC10899115; doi:10.1038/s43018-023-00703-y)

Figure 5m. Western blots from FC1245 sgCtrl or sgEnpp2 nuclear cell lysates upon treatment with 40ng/ml Il-4 and TNF a

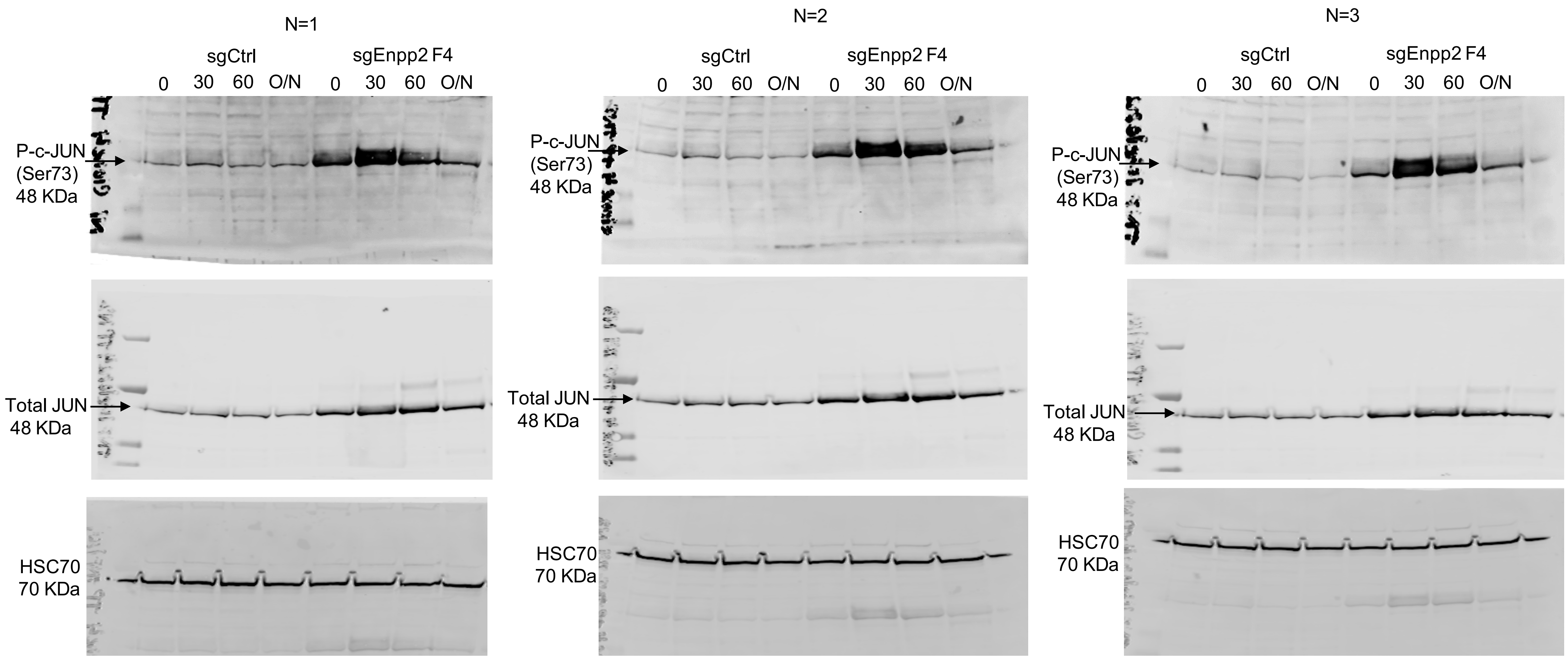

Supplement: Supplementary file 9 — Unprocessed western blots and/or gels. [file 43018_2023_703_MOESM9_ESM.pdf]
